# Molecular-guided therapy predictions reveal drug resistance phenotypes and treatment alternatives in malignant peripheral nerve sheath tumors

**DOI:** 10.1186/1479-5876-11-213

**Published:** 2013-09-17

**Authors:** Jacqueline D Peacock, David Cherba, Kevin Kampfschulte, Mallory K Smith, Noel R Monks, Craig P Webb, Matthew Steensma

**Affiliations:** 1 Van Andel Research Institute, Grand Rapids, Michigan, USA; 2 Hope College, Holland, Michigan, USA; 3 Spectrum Health Medical Group, Grand Rapids, Michigan, USA; 4 Michigan State University College of Human Medicine, East Lansing, Michigan, USA

## Abstract

**Background:**

Malignant peripheral nerve sheath tumors (MPNST) are rare highly aggressive sarcomas that affect 8-13% of people with neurofibromatosis type 1. The prognosis for patients with MPNST is very poor. Despite TOP2A overexpression in these tumors, doxorubicin resistance is common, and the mechanisms of chemotherapy resistance in MPNST are poorly understood. Molecular-guided therapy prediction is an emerging strategy for treatment refractory sarcomas that involves identification of therapy response and resistance mechanisms in individual tumors. Here, we report the results from a personalized, molecular-guided therapy analysis of MPNST samples.

**Methods:**

Established molecular-guided therapy prediction software algorithms were used to analyze published microarray data from human MPNST samples and cell lines, with benign neurofibroma tissue controls. MPNST and benign neurofibroma-derived cell lines were used for confirmatory in vitro experimentation using quantitative real-time PCR and growth inhibition assays. Microarray data was analyzed using Affymetrix expression console MAS 5.0 method. Significance was calculated with Welch’s t-test with non-corrected p-value < 0.05 and validated using permutation testing across samples. Paired Student’s t-tests were used to compare relative EC50 values from independent growth inhibition experiments.

**Results:**

Molecular guided therapy predictions highlight substantial variability amongst human MPNST samples in expression of drug target and drug resistance pathways, as well as some similarities amongst samples, including common up-regulation of DNA repair mechanisms. In a subset of MPNSTs, high expression of *ABCC1* is observed, serving as a predicted contra-indication for doxorubicin and related therapeutics in these patients. These microarray-based results are confirmed with quantitative, real-time PCR and immunofluorescence. The functional effect of drug efflux in MPNST-derived cells is confirmed using in vitro growth inhibition assays. Alternative therapeutics supported by the molecular-guided therapy predictions are reported and tested in MPNST-derived cells.

**Conclusions:**

These results confirm the substantial molecular heterogeneity of MPNSTs and validate molecular-guided therapy predictions in vitro. The observed molecular heterogeneity in MPNSTs influences therapy prediction. Also, mechanisms involving drug transport and DNA damage repair are primary mediators of MPNST chemotherapy resistance. Together, these findings support the utility of individualized therapy in MPNST as in other sarcomas, and provide initial proof-of concept that individualized therapy prediction can be accomplished.

## Background

Malignant peripheral nerve sheath tumors (MPNSTs) are aggressive sarcomas associated with substantial morbidity and mortality [1]. MPNSTs are rare in the general population, affecting about 1 in 100,000 people each year [2], whereas individuals with neurofibromatosis type 1 (NF1) carry an 8-13% lifetime risk of developing an MPNST [1]. Despite aggressive, multi-modal treatment, overall survival is poor for both primary and metastatic MPNST [1,3].

Chemotherapy resistance is a hallmark of both primary and recurrent MPNSTs [4,5] owing to a variety of factors, most notably up-regulation of drug efflux transporters [4,6-8]. Alternative mechanisms of chemotherapy resistance in MPNSTs and other sarcomas have been described, including Twist 1 overexpression [9], Bcl-xl overexpression [10], and autophagy induction [11]. Escalation of DNA repair processes is also observed in other chemotherapy-resistant sarcomas [12-14]. The doxorubicin target, topoisomerase II (TOP2A), is significantly overexpressed in MPNSTs [15] compared to neurofibromas [16]. Doxorubicin binds to the topoisomerase II complex following DNA strand breaks, interrupting cellular replication [17]. However, overexpression of TOP2A is associated with diminished survival in MPNST, confirming that overexpression of the doxorubicin target is insufficient to overcome established mechanisms of doxorubicin resistance [15]. Doxorubicin-based chemotherapy regimens are typically used to treat MPNST, but the therapeutic benefit is modest and closely parallels that of other soft-tissue sarcoma regimens [18,19], and dose limiting toxicity is common [20].

The refractory nature of MPNSTs is attributable to a high degree of molecular heterogeneity, both in terms of mechanisms underlying disease progression [21] and rapidly evolving therapy resistance. Studies confirm deletion or loss of function in tumor suppressor genes, including NF1, HMMR/RHAMM, TP53, and duplications or gain of function mutations in several oncogenes, including MET, HGF, EGFR, ITGB4, and PDGFRA [22]. Other deregulated pathways in MPNSTs include a variety of well-characterized drug targets such as mTOR, HGF/Met, TOP2A, Ras, and steroid hormones [15,16,22-27].

Molecular-guided therapy prediction or personalized medicine (PMED) strategies are currently under evaluation for use in recurrent and refractory pediatric brain tumors (NCT01802567), neuroblastoma (NCT01355679) and sarcomas (NCT01772771). This approach is also a promising treatment alternative for therapy-resistant cancers like MPNST [28-30]. PMED workflows follow a knowledge and rules-based statistical algorithm that converts genomic profiling data into an ordinal ranking of therapies. Drug predictions are therefore agnostic to disease context and adaptable to a variety of clinical scenarios. Essential to the PMED drug prediction algorithm is the reconciliation of predicted therapies selected from a comprehensive drug list against known mechanisms of chemotherapy resistance and drug resistance biomarkers. This knowledge-based rules approach relies on databases, such as DrugBank, that feature annotated references to over one thousand drugs and target molecules. PMED platforms also feature topological analysis tools which identify drug targets and potential mechanisms of resistance based on gene network perturbation. This approach is complementary to a single gene interrogation and allows for a broader systems-based analysis of disease-specific molecular pathogenesis (GeneGo-Thomson Reuters) [31-35]. While the clinical efficacy of PMED approaches is still under investigation, the PMED bioinformatics approach is a robust tool for discovery-level research into the molecular pathogenesis of MPNSTs.

Here, we present data supporting the PMED strategy as a useful method for determining mechanisms of chemotherapy resistance and identifying potential alternative therapeutics in individual MPNSTs. The use of benign precursor neurofibromas as a biologically relevant control in the PMED analysis is novel and provides insight into the genomic alterations underlying conversion from neurofibroma to MPNST. We also demonstrate that novel predicted therapies have *in vitro* efficacy against highly drug resistant MPNST-derived cells [35].

## Methods

### Microarray data

Microarray data on MPNST samples, neurofibromas, and MPNST-derived cell lines were accessed via NCBI Gene Expression Omnibus (GEO) repository [36] as indicated in text. Additional benign neurofibroma samples were acquired through an established tissue collection initiative in collaboration with Spectrum Health. All specimens were obtained according to an IRB approved protocol within Spectrum Health. Affymetrix U133 2.0 plus chip arrays were performed at Clinical Research Laboratories (CRL, Lenexa, KS). Purified RNA was used for the preparation of amplified cDNA (NuGen Ovation Pico WTA System). Amplified cDNA was then fragmentated and labelled (NuGen Encore Biotin Module) and hybridized to GeneChip Human Genome U133 Plus 2.0 Array (GeneChip® Hybridization, Wash and Stain Kit, Affymetrix). The arrays were scanned by using GeneChip Scanner 3000 7G and the intensity files were analyzed by Expression Console Software. Array data was normalized using Affymetrix expression console MAS 5.0 method and further filtered to remove probes with absent calls and expression intensities less than 100 in over 40% of samples. Differentially expressed genes were identified using Welch’s t-test with non-corrected p-value < 0.05 and validated using permutation testing across samples. Most significant probe sets of top 100 and top 200 probes were submitted to GeneGo for extensive network and pathway enrichment analysis. Heat maps were generated using XenoBase® version 3.5 from Affymetrix array data using MAS 5.0 normalization. Clustering was performed in both sample and probe dimensions using average linkages with a Pearson correlation distance metric.

### Molecular-guided personalized medicine (PMED) analysis

**Figure 1:**
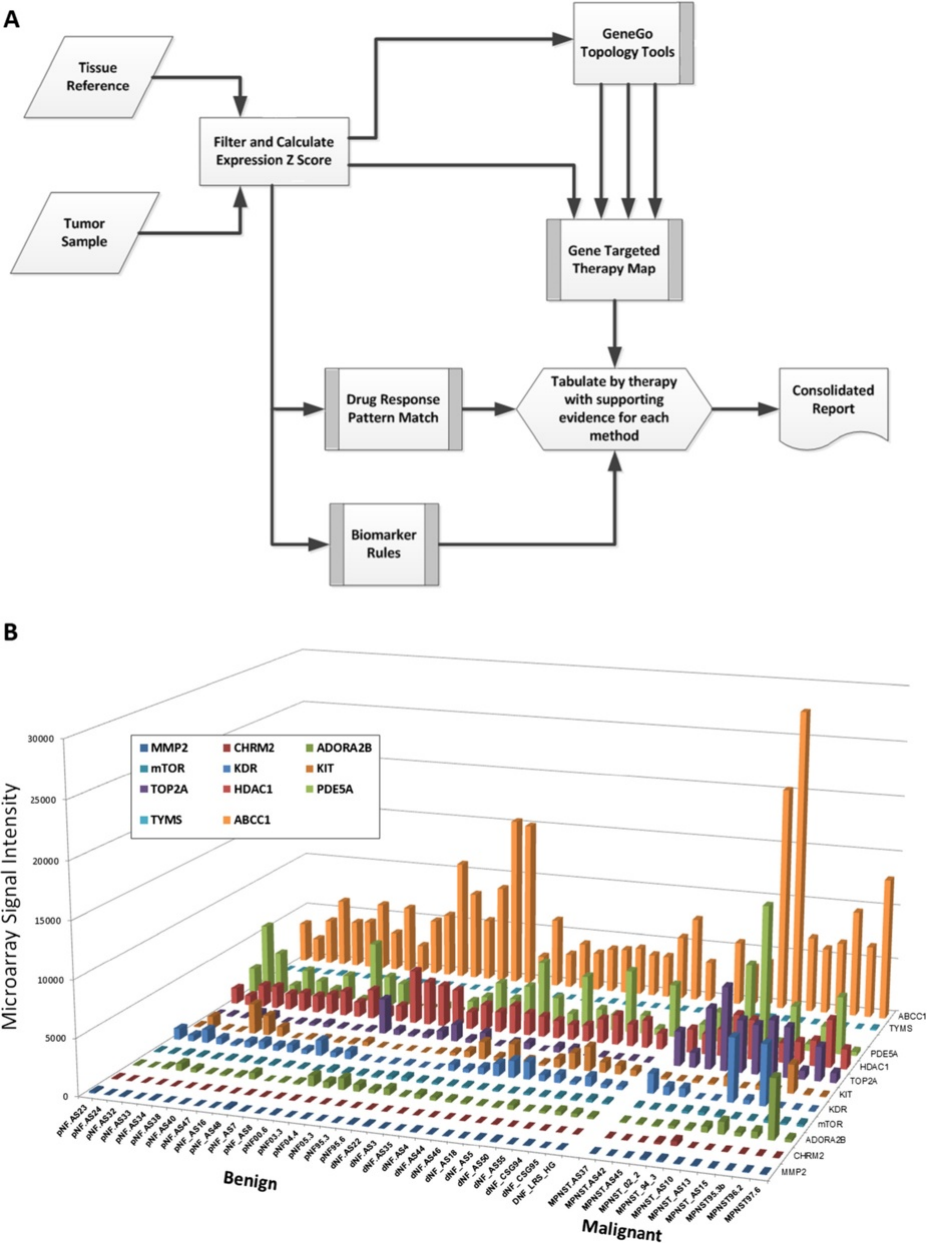
**Molecular-guided therapy prediction process. A)** Each MPNST sample is compared to a benign tumor tissue reference pool and processed as shown. Consolidated reports include individual rankings of therapeutics for each sample (see Table 1 and Additional file 2). **B)** Summary graph depicting microarray signal intensity for transcripts contributing to molecular-guided therapy predictions in MPNST samples in comparison with benign neurofibromas. Additional graphs for increased visibility of individual results are also provided in Additional file 4.

For each individual tumor sample tested, microarray data from a single sample was compared to pooled benign controls. This process was performed for a total of 15 samples including MPNST and MPNST-derived cell lines (public dataset), and neurofibroma tissue samples. Microarray data processed as above was analyzed using XenoBase-based analysis software, a molecular-guided therapy prediction methodology and reporting tool developed at the Van Andel Research Institute [34,35]. Tumor gene expression levels from Affymetrix U133 2.0 plus chip were normalized using MAS 5.0 Affymetrix expression console and compared to a benign tumor reference set. Relative expression intensities were converted to Z-score values and the gene list with significant expression deviation from the reference set are supplied directly to the Gene Targeted Therapy Map [37] as well as to the GeneGo Topology tools [34] that identify additional significant genes implied by topological analysis. Topologically identified genes were also supplied to the Gene Targeted Therapy Map. Z-score expression values were also supplied to two drug response pattern evaluation methods, PGSEA [38] and CMAP [39]. PGSEA and CMAP score the expression pattern against known response to therapy and suggest possible effective therapies. The final method to supply therapy choices is driven by expression levels and applied to specific biomarker rules based on strong evidence from clinical trial work that validates the biomarkers for both indicated and contra-indicated therapies [32,40]. All MPNST and MPNST-derived sample data, in addition to data from benign samples for which paired tumor-derived cell lines, RNA, and histology were available for future use were individually analyzed using this process. Finally, results from these analyses are integrated and ranked according to summary scores. A diagram of this process is provided in Figure 1A, and a more thorough description is provided as Additional file 1.

### Quantitative real-time PCR

Microarray data was confirmed using real-time polymerase chain reaction (qRT-PCR). Total RNA was extracted from cultured MPNST cell lines and benign neurofibroma-derived cell lines during logarithmic cell growth using TRIzol reagent (Invitrogen). Neurofibroma cell lines were derived from benign neurofibromas using established protocols [41]. Synthesis of cDNA was performed using 500 ng of RNA according to manufacturer’s instructions (High Capacity cDNA Reverse Transcription Kit, Invitrogen). Primers used for qRT-PCR were as follows *ABCC1*-Forward (F), GAGGAAGGGAGTTCAGTCTT; *ABCC1*-Reverse (R), ACAAGACGAGCTGAATGAGT; *ABCC3*-F, CACACGGATCTGACAGACAATGA; *ABCC3*-R, ACAGGGCACTCAGCTGTCTCA; *ABCC4*-F, TGTGCTTTTTAAGGCTTCACTCAAT; *ABCC4*-R, TTGTCCTTCGTATAGCAAGTTTTTTG; *ABCC5*-F, GAGAACCAGCACTTCTGGGA; *ABCC5*-R, TGAGCTGAGAATGCATGGAG; *ABCC6*-F, AAAGTACACACAGCATGGCAGTTC; *ABCC6*-R64, GCTCCCGGCTAGACCCTTAA; *ABCG5*-R232, GTTCACATACACCTCCCCCA; *ABCG5*-F101, TCCTTGTACGTGGAGAGCG; *GAPDHF*, TGGTATCGTGGAAGGACTCATGAC; *GAPDHR*, TGCCAGTGAGCTTCCCGTTCAGC. Reactions were performed in duplicate at 10 μl volume using Sybr Select master mix (Applied Biosystems) according to manufacturer’s instructions. Melt curve analyses are performed following all reactions to ensure detection of a single product based upon single and consistent melting temperatures for each primer set using StepOne Software v2.3 (Applied Biosystems) standard parameters. Data is normalized using *GAPDH* expression and represented as fold change relative to a control sample (2^ΔΔC_T_) as indicated in the respective results.

### Immunofluorescence

Cells grown on 8-well chamber slides (Nunc) were fixed in 4% paraformaldehyde, blocked in PBS with 10% goat serum, and incubated in primary antibodies against ABCC1 (Abcam ab24102) and S100 (Dako Z0311) at 1:50 and 1:400 dilution, respectively, overnight at 4°C. Cells were washed in PBS, and secondary incubations were conducted for 45 minutes at room temperature with respective Alexa Fluor-488 Donkey anti-Mouse IgG and Alexa Fluor-568 Donkey anti-Rabbit IgG secondary antibodies at 1:400 dilution. Slides were mounted in Vectashield with DAPI (Vector Labs) for nuclear counterstaining. All images were obtained using identical acquisition settings with 60× objective on an A1 confocal Ti microscope (Nikon).

### Growth inhibition experiments

MPNST-derived cell lines NF96.2, NF02.2, and NF94.3 (ATCC) and benign neurofibroma cell lines were maintained in 5% CO_2_ at 37C, in modified DMEM with 10% fetal bovine serum and 1% penicillin/streptomycin. Growth inhibition experiments were carried out in DMEM supplemented with 10% FBS in 96-well plate format. Cells were seeded at 2×10^3^ cells per well and allowed to attach for 24 hours prior to drug treatment for 96 hours. Doxorubicin (LC Laboratories) dosages included 5 μg/ml, 2.5 μg/ml, 1.25 μg/ml, 625 ng/ml, 312 ng/ml, 156 ng/ml, 78 ng/ml, 40 ng/ml, 20 ng/ml, and 10 ng/ml. Vorinostat, rapamycin, and etoposide (LC Laboratories), as well as thalidomide (Sigma), were used at doses ranging from 2 mM to 100 nM. Freshly prepared verapamil (Sigma) was added at 100 μM where indicated. Trichloroacetic acid fixation and sulforhodamine B (SRB) staining was performed as described [42] as a surrogate cell count measurement. EC_50_ was defined as the drug concentration causing a 50% reduction in net signal versus untreated controls as interpolated from line of best fit. An EC_50_ was calculated for each individual experiment (n = 5) and Student’s t-test was used to compare EC_50_ from doxorubicin only treatments to verapamil (100 μM) plus doxorubicin.

## Results

### Molecular-guided therapy predictions

Molecular-guided therapy prediction analyses (Figure 1A) were performed based on published expression data from five MPNST-derived cell lines and six human MPNST tissue samples. This analysis identified hypothetical drug targets, indicators of drug sensitivity, and indicators of drug resistance or insensitivity using curated biomarker rules, drug response knowledge, and topology tools [36]. Scores based on a synthesis of this information are assigned to each drug and drugs are ranked in a consolidated summary report. Reports were also generated in this way for benign neurofibroma data, using normal nerve tissue as a reference. Table 1 includes a truncated summary of drug recommendations and gene expression contributing to the top ranked drugs for each tumor. Complete records of the summary drug recommendations and results of intermediate analyses are reported in Additional files 2 and 3. Microarray-based expression levels of the major transcripts contributing to drug responsiveness and drug resistance predictions for each MPNST sample are shown in Figure 1B, with additional detail provided in Additional file 4.

**Table 1:** Top 3 drugs indicated by personalized medicine analysis for MPNST and neurofibroma samples

**Sample**	**Drug 1**	**Related transcripts**	**Drug 2**	**Related transcripts**	**Drug 3**	**Related transcripts**
*MPNST-derived cell lines*						
**NF02.2**	vorinostat	*HDAC1-4, 6*	teniposide	*TOP2A*	sirolimus	*MTOR, FKBP1A*
**NF94.3**	pravastatin	*MMP2, MMP14, TIMP2*	flavoxate	*CHRM2*	sildenafil	*PDE5A*
**NF96.2**	vorinostat	*HDAC1,2, 6*	chlorpromazine	*HTR7, DRD2,*	dasatinib	*ABL1, FYN, KIT*
*MPNST samples*						
**AS10**	pazopanib	*KDR, FLT1, PDGFRB*	sorafenib	*KDR, FLT1, PDGFRB*	sunitinib	*KDR, FLT1, PDGFRB*
**AS13**	teniposide	*TOP2A*	etoposide	*TOP2A*	doxorubicin	*TOP2A*
**AS15**	teniposide	*TOP2A*	vorinostat	*HDAC2*	sunitinib	*KDR, CSF1R*
**AS37**	doxycycline	*MMP9, MMP13*	pravastatin	*MMP9, MMP13*	vorinostat	*HDAC2,3,4*
**AS42**	octreotide	*SSTR2*	doxycycline	*MMP9, MMP13*	pravastatin	*MMP9, MMP14*
**AS45**	teniposide	*TOP2A*	octreotide	*SSTR2*	vorinostat	*HDAC2,3*
*Benign neurofibroma samples*						
**MS37T**	dasatinib	*EPHA2,KIT*	nilotinib	*KIT*	pazopanib	*KIT,KDR*
**MS90T**	biperiden	*CHRM1*	carbinoxamine	*CHRM1*	clozapine	*CHRM1*
**MS135T**	dasatinib	*EPHA2*	sorafenib	*KIT*	pazopanib	*KIT,KDR*
**MS142T**	fluticasone	*PGR*	medroxyprogesterone	*PGR*	sorafenib	*FLT3*
**MS153T**	dasatinib	*EPHA2,KIT,LCK,SRC*	imatinib	*KIT*	nilotinib	*KIT*
**MS156T**	sorafenib	*KIT*	sunitinib	FLT3	clofarabine	*POLA1*

As expected, *TOP2A* overexpression is observed in nearly all MPNST and MPNST-derived samples, favoring doxorubicin and other TOP2A inhibitors based on drug target expression (Figure 1B). Variable expression of other drug-targetable pathways is also observed, including *mTOR* (rapamycin)*.* In several samples, high *ABCC1* expression is apparent (Figure 1B) and is highlighted by the molecular-guided therapy analysis as a hypothetical doxorubicin resistance mechanism. *TYMS* overexpression, also observed, has been shown by others to correlate with doxorubicin resistance phenotypes as well [43,44]. Re-analysis of the published [36] microarray dataset confirms that *ABCC1* is the most highly expressed ABC transporter significantly elevated in MPNSTs relative to benign plexiform neurofibromas (Additional file 5). Other members of the ABCC family are also elevated in the MPNSTs as a group, including *ABCC3, ABCC4,* and *ABCC6.*

**Figure 2:**
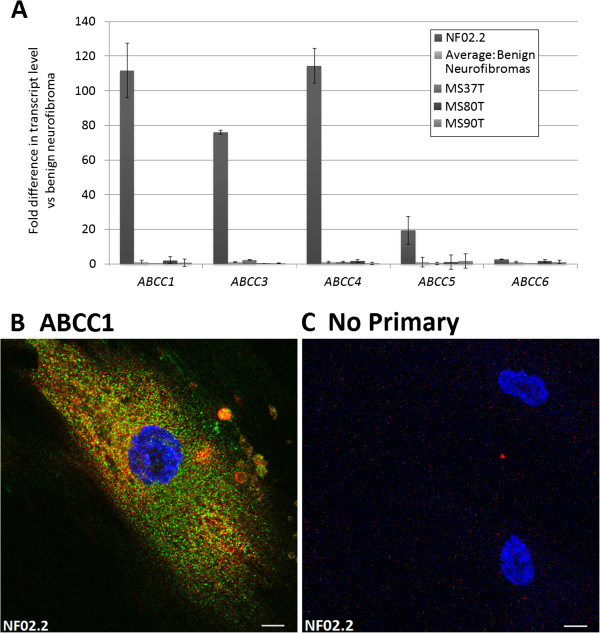
**ABCC1 expression in MPNST-derived cell line NF02. A)** Quantitative real-time PCR confirms elevated expression in NF02.2 compared to benign neurofibroma-derived cell lines MS37T, MS80T, and MS90T. **B)** Immunofluorescent staining for ABCC1 (green) and S100 (red) with DAPI (blue) nuclear stain as indicated. No primary antibodies were added in **(C)**. Scale bar = 10 μm.

NF02.2, an MPNST-derived cell line (ATCC [45]) showed significant and consistent expression of *ABCC1.* Quantitative real-time PCR confirms the high level of expression of *ABCC1* in the NF02.2 cell line relative to benign neurofibroma-derived cells and other ABCC family members (Figure 2A). ABCC1 protein is also detectable by immunofluorescent staining in NF02.2 cells in culture (Figure 2B).

### Function and expression of ABC transporters *in vitro*

**Figure 3:**
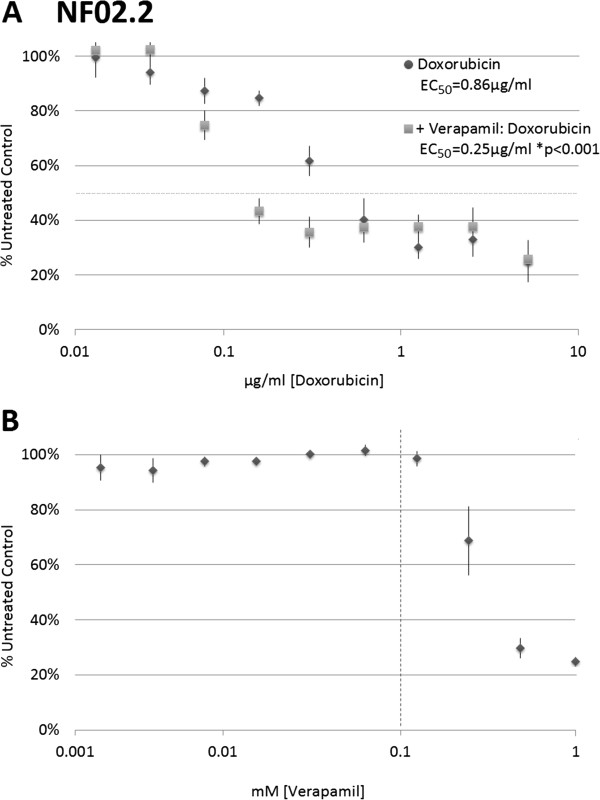
**Doxorubicin-mediated growth inhibition in MPNST-derived NF02.2 cells with high ABCC1 expression. A)** Relative cell content (as a percentage of untreated control cells) following 96 hours of growth in doxorubicin-containing media with and without 100 μM verapamil. Average doxorubicin EC_50_ 1.23 μg/ml, versus 0.21 μg/ml in the presence of verapamil *p = 0.003, n = 5. **B)** No growth inhibition is observed with verapamil alone at concentrations 125 μM and lower. Dotted line indicates 100 μM concentration used in **(A)**.

In order to examine the functional relevance of ABCC1 and ABC family drug transporter activity, growth inhibition assays were performed using a broad range of doxorubicin dosages (5 μg/ml [8.6 μM] to 20 ng/ml [34 nM]) in the presence or absence of 100 μM verapamil, a calcium channel blocker that inhibits ABC transporter activity. Significantly lower doxorubicin EC_50_ values are obtained when doxorubicin dose is combined with verapamil (0.861 μg/ml ± 0.17 versus 0.248 μg/ml ± 0.07, p = 0.0002 with paired Student’s t-test, Figure 3A). Low-dose (<125 μM) verapamil alone does not affect growth (Figure 3B).

**Figure 4:**
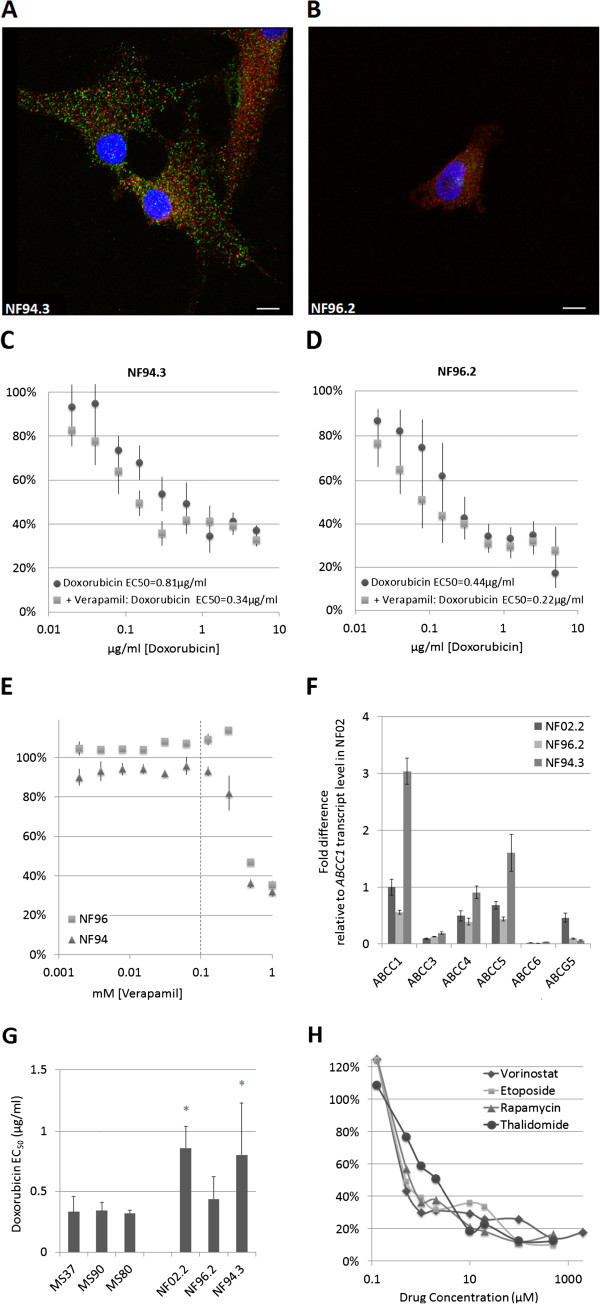
**ABCC1 expression and transporter function in MPNST-derived cell lines NF94.3 and NF96.2.** Immunofluorescent staining for ABCC1 (green) and S100 (red) with DAPI (blue) nuclear stain as indicated in NF94.3 **(A)** and NF96.2 **(B)**. Scale bar = 10 μm. **C)** Relative cell content following 5 days of growth in doxorubicin-containing media with and without 100 μM verapamil in NF94.3 cells. Average doxorubicin EC_50_ 0.81 μg/ml, versus 0.34 μg/ml in the presence of verapamil (*p = 0.025, n = 5). **D)** NF96.2 cells; average doxorubicin EC_50_ 0.44 μg/ml, versus 0.22 μg/ml in the presence of verapamil (n.s. p = 0.16, n = 5). **E)** No growth inhibition with verapamil alone at concentrations 125 μM and lower. Dotted line indicates 100 μM verapamil concentration used in **(C, D)**. **F)** Quantitative real-time PCR for ABCC family transporter transcript levels. **G)** Doxorubicin EC_50_ values for benign neurofibroma-derived cell lines as compared to MPNST-derived cell lines. **H)** Growth inhibition curves for a representative experiment with molecular-guided therapy predicted drugs. Data is graphed as percentage untreated control per drug concentration.

Two additional MPNST cell lines, NF94.3 and NF96.2, are also examined. In NF94.3, similar to NF02.2, high *ABCC1* expression is highlighted by the molecular-guided therapy analysis as a hypothetical doxorubicin resistance mechanism, whereas NF96.2 is not flagged for high *ABCC1* expression. ABCC1 is detectable by immunofluorescence in NF94.3 (Figure 4A) but not NF96.2 (Figure 4B). A small effect of verapamil channel blockade on doxorubicin EC_50_ is observed in NF94.3 cells (0.81 μg/ml ± 0.42 versus 0.34 μg/ml ± 0.20, p = 0.025 paired Student’s t-test, Figure 4C), while no significant effect is observed in low-*ABCC1* expressing NF96.2 cells (0.44 μg/ml ± 0.31 versus 0.22 μg/ml ± 0.25, p = 0.16 paired Student’s t-test, Figure 4D). No effect is observed for verapamil-only treatments at concentrations below 125 μM in either cell line (Figure 4E).

#### Microarray analysis of drug transport gene expression

**Figure 5:**
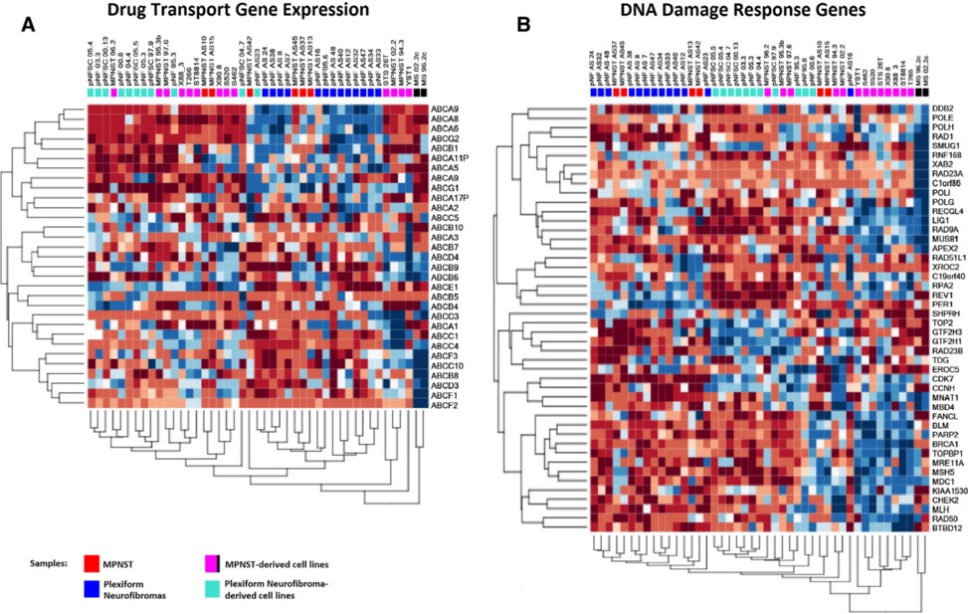
**Drug transporter and DNA damage response gene expression in MPNSTs and benign neurofibromas. A)** Heat map detailing unsupervised clustering of MPNSTs, MPNST-derived cell lines, plexiform neurofibromas and derived cell lines using a drug-transport related gene list. **B)** Clustering using a DNA damage response gene list. Two MPNST-derived cell lines (Black boxed samples) were repeated on a separate microarray study at higher passage.

In addition to ABC transport, other mechanisms of drug resistance are undoubtedly present in MPNSTs. Additional microarray analysis revealed activation of DNA damage repair processes that may contribute to insensitivity to doxorubicin-mediated DNA damage. In contrast to drug transport gene expression, which is highly variable amongst MPNSTs (Figure 5A), DNA damage repair and related pathway gene expression is consistently higher in MPNSTs and MPNST-derived cell lines when compared to benign, plexiform neurofibromas (Figure 5B). DNA damage repair processes are also elevated in MPNST-derived cell lines when compared to the tumors themselves. Therefore, this effect may be exaggerated by or selected for during the tissue culture process. Significant changes in other mechanisms of drug resistance, however, were not observed in our analysis. Autophagy, Twist1, and apoptosis related signaling were not among significantly altered gene ontology processes (data not shown).

## Discussion

Our results demonstrate that molecular-guided therapy predictions can be used to identify systematic patterns of drug resistance in MPNSTs based on analysis of human MPNST samples when compared to benign neurofibroma precursors. Significant molecular heterogeneity amongst MPNSTs is observed, and the functional consequences of this are examined *in vitro.* ABCC transporters are highly overexpressed in some samples, and transporter activity appears to play a modest but significant role in decreasing doxorubicin effectiveness in this subset of cultured MPNST-derived cells. Although transporter inhibitors have not yet shown clinical utility [46,47], new agents targeting this important resistance mechanism are currently under investigation [48].

Considering only the list of current FDA approved drugs, however, we have also identified alternative therapeutics that may be effective in these drug-resistant patients using our molecular-guided therapy analysis. This analysis synthesizes biomarker, network, and drug target based predictions for each individual tumor sample by comparing the tumor to benign controls. The top three drugs predicted for each cell line and tumor studied are listed in Table 1. The top four alternative therapeutics for the doxorubicin-insensitive NF02.2 cell line were vorinostat, etoposide/teniposide, sirolimus, and lenalidomide. However, many previous studies have demonstrated cross-resistance to doxorubicin and etoposide or teniposide, so these are likely not meaningful alternatives in doxorubicin-refractory tumors [49-51]. Vorinostat, an HDAC inhibitor, is suggested for use in NF02.2 cells based on drug response signature, network target activity (including elevation of *HDAC1, 2, 3*, and *6*), and drug target expression (elevated *HDAC2*) evidence. Sirolimus (rapamycin) is suggested due to elevated drug target (*mTOR*) expression and pathway signaling. Elevated mTOR activity has been observed previously in MPNSTs and neurofibromas and is currently the subject of multiple clinical trials (NCT00634270, NCT01661283, NCT00652990) [52,53]. Lenalidomide, a derivative of thalidomide, was suggested for use based on elevated *PTGS2* and *TNF* expression (see Additional file 2) [54-56].

Additionally, we examined the efficacy of these predicted therapeutics in NF02.2 cells *in vitro.* Our results demonstrate efficacy at low μM concentrations for rapamycin (Sirolimus) [1.63 μM ± 0.26] and vorinostat [2.57 μM ± 0.88]*.* EC_50_ values for etoposide [16.2 μM ± 5.92] and thalidomide [34.72 μM ± 25.13] are relatively higher (n = 4; one representative experiment is shown in Figure 4H), but deserve further examination in combination with cytotoxic agents.

Notably, drug transport expression is highly variable between MPNSTs and does not fully account for the observed therapy resistance. Our additional analysis highlighted DNA damage repair gene expression as a possible chemotherapy resistance mechanism. DNA damage repair pathways are significantly elevated in MPNSTs as a group. This implies an elevated resistance to DNA damaging cytotoxic chemotherapy agents, including doxorubicin, and consideration should be made to routinely include elevation in DNA damage repair pathway gene expression in future molecular-guided therapy prediction analyses.

## Conclusions

Here, we provide evidence that the impact of patient heterogeneity and drug transporter expression must be considered in the selection of alternative treatment strategies for treatment refractory MPNST patients. We also confirm that PMED-predicted therapies have potential activity against MPNSTs. Future studies should focus on validating individualized drug predictions *in vivo,* improving identification of effective drug combinations, and expanding strategies to leverage PMED tools in discovery-level research.

### Availability of supporting data

Microarray data for this study are deposited with the GEO repository: GSE50208.

## Abbreviations

MPNST: Malignant peripheral nerve sheath tumor; NF1: Neurofibromatosis type 1; qRT-PCR: Quantitative real-time polymerase chain reaction; PBS: Phosphate buffered saline; DAPI: 4',6-diamidino-2-phenylindole; ATCC: American type culture collection; EC50: Drug concentration causing a 50% reduction in net signal (cell content) versus untreated controls.

## Competing interests

CPW is co-inventor of the XenoBase® bioinformatics platform that has been licensed to TransMed Systems. The authors declare no other competing interests.

## Authors' contributions

JDP participated in study design and coordination, data analysis, carried out in vitro studies including growth inhibition assays, immunofluorescence and confocal microscopy, and drafted the manuscript. MKS and KK contributed to growth inhibition assays, data analysis and contributed to the manuscript. DC performed microarray analysis, molecular-guided therapy predictions, data analysis, and contributed to the manuscript. MS, NM, and CPW participated in the conception and design of the study and reviewed the manuscript. All authors read and approved the final manuscript.

## Supplementary Material

Additional file 1 **Description of the molecular-guided therapy prediction process.** Additional detail is provided in this document to better describe the data flow, reference selection, drug knowledge database, drug target expression analysis, topological methods, drug response signatures, drug sensitivity signatures, and method variance involved in the molecular guided therapy predictions. Click here for file

Additional file 2 **Detailed summary reports of molecular-guided therapy predictions.** A summary spreadsheet is provided detailing the summary results for the analyzed MTTB neurofibroma samples (A-G) and MPNSTs from the public data set (A’-G’). Ranked therapeutics and corresponding scores as indicated for each sample (A) are based upon intermediate results from B) drug target expression, C) network topology and target activity, D) parametric gene set enrichment analysis (PGSEA), E) connectivity map (CMAP) analysis, F) biomarker based rules (resistant) analysis, and G) biomarker rules (sensitive) analysis. Click here for file

Additional file 3 **Detailed summary reports of molecular-guided therapy predictions.** Summary therapy predictions, drug target (GeneGoDrugTarget) expression, cumulative Network topology results, parametric gene set analysis (PGSEA), connectivity map (CMAP), and biomarker-based rules (BiomarkerResistant/BiomarkerSensitive) in a CSV file for greater data accessibility. This file supplies the same data as Additional file 2. Click here for file

Additional file 4 **Relative expression of transcripts related to therapeutic responsiveness scores in the personalized medicine analysis.** This figure is an expansion of data presented in Figure 1 for improved clarity. Normalized signal intensity is graphed for *ABCC1, ADORA2B, CHRM2, HDAC1, KDR, KIT, MMP2, mTOR, PDE5A, TOP2A*, and *TYMS* with average intensity from benign neurofibromas presented for comparison to the individual MPNST samples as indicated. Click here for file

Additional file 5 **Relative expression of ABC transporter family genes by microarray.** Normalized signal intensity is graphed for all probes for *ABCC, ABC1, ABCB*, and *ABCD-G* family transcript expression. Significantly increased transcript levels in MPNSTs compared to plexiform neurofibromas are indicated (*) for p < 0.05. Click here for file
