# Supplementary material for: Molecular-guided therapy predictions reveal drug resistance phenotypes and treatment alternatives in malignant peripheral nerve sheath tumors
Source: J Transl Med. 2013 Sep 17;11:213. doi: 10.1186/1479-5876-11-213 (PMC3848568; doi:10.1186/1479-5876-11-213)
Supplement: Additional file 1 — Description of the molecular-guided therapy prediction process. Additional detail is provided in this document to better describe the data flow, reference selection, drug knowledge database, drug target expression analysis, topological methods, drug response signatures, drug sensitivity signatures, and method variance involved in the molecular guided therapy predictions. [file 1479-5876-11-213-S1.docx]

# Molecular Guided Therapy: Personalized Medicine

Gene expression levels from individual tumor samples were obtained using Affymetrix U133 2.0 plus chip data and normalized using MAS 5.0 Affymetrix expression console. Each sample is compared to a tissue reference set. The relative expression intensities are converted to Z-score values with respect to the reference set. Gene lists with significant expression deviation from the reference set are supplied directly to the Gene Targeted Therapy Map [[1](#_ENREF_1)] as well as to the GeneGo Topology tools [[2](#_ENREF_2)] that identify additional significant genes implied by topological analysis. These genes indicated as significant from the topological tool are also supplied to the Gene Targeted Therapy Map. Z-score expression values are also supplied to two drug response pattern evaluation methods. PGSEA [[3](#_ENREF_3)] and CMAP [[4](#_ENREF_4)] score the expression pattern against known response to therapy and suggest possible effective therapies. The last method used to suggest therapy choices is driven by expression levels and applied to specific biomarker rules [[5](#_ENREF_5), [6](#_ENREF_6)]. These rules are based on strong evidence from clinical trial work that validates the biomarkers for both indicated and contra indicated therapies.

## Reference selection

There are several different logical choices for the reference set given specific conditions. The most desirable is a reference set consisting of tissue specific normal samples which would highlight the differences between tumor and normal. As extensive sets of tissue-specific normal samples are limited, several alternatives exist. A broad scope reference consists of a collection of normal tissue and tumors which capture a large variance. Comparing samples to this broad scope reference is expected to highlight genes that are very significant in deviant expression. The third common reference set consists of a whole body normal set which attempt to capture the significant variance of tissue specific genes. In this study, pooled benign neurofibroma data were used as a reference set for the individual MPNST samples, and neurofibroma-adjacent normal nerve tissue data were used as a reference set for the benign neurofibroma samples. Although neurofibromas are not normal tissue, they are a benign precursor lesion to MPNST, and were therefore selected to help highlight the changes that occur in the malignant transformation process.

## Drug Knowledge Database

Information for the Drug Knowledge Database was tabulated from a variety of sources. The key components of this database include FDA approved drugs that have documented gene targets, biomarker indications of effectiveness, and/or complex gene expression patterns that indicate effect or response. This database is updated as new information is validated. The additional information contained in GeneGo about gene interactions is maintained independently and is used to perform the topological analysis.

This database has five sections. The first is an index of drugs that inhibit specific gene expression. A drug can inhibit more than one gene so that a drug therapy suggestion can originate from multiple gene expression values. The drug target expression algorithm and the results from topological analysis use this section of the database to map gene expression to drug suggestions.

The next two sections are associated with the pattern matching provide by PGSEA and CMAP. Both of these methods have a larger selection of drugs suggestion than are utilized by the personalized medicine therapy suggestion process. The list for both methods was restricted to the FDA approved list and also only those which have some published indication of effectiveness. Lists including drugs not yet approved by the FDA are also available, but were not used in this study.

The final two components of the analysis consider biomarker rules that use gene expression to invoke the rule. The rules have both contra-indicated (resistant) and indicated (sensitive) content. The tumor’s gene expression level is compared to an established limit and if the limit is exceeded then the rule is invoked. The “resistant” rules can contra-indicate drugs that are expected to have no effect or drugs that, under these conditions, have known adverse effects. The “sensitive” rules can indicate drugs where there is not a direct connection between the drug’s target gene and the known effectiveness.

## Drug Target Expression

The majority of drugs with known gene targets inhibit expression of the gene or gene products. Therefore the suggestion of possible effective therapies is based on over expression of a probe compared to the reference set probe. All comparisons are done at probe level so multiple probes can indicate a given gene. The basic algorithm identifies any probe for a drug target gene where the z-score is greater than three. The list of drugs associated with the probe-gene is generated, with the score derived by the negative log of the p-value associated with the z-score.

## Topological Methods

There are three types of topological methods: convergence, divergence, network drug target method. All three methods are provided with the same basic set of information in the form of an Entrez gene list. This list is includes all the genes that have a z-score greater than or equal to two or the top 500 if more than 500 genes have a z-score greater and equal to two.

The convergence topological method identifies genes in the interactome knowledge base that are implicated by the submitted gene list as having significantly enrichment for convergence in the shortest path analysis between all the genes in the submitted list. This implies that an inhibitor of that gene’s expression may have significant overall effect its pathway and therefore on the over expressed gene list. The gene indicated in “Network” results presented here as Additional File 2, therefore, are genes where expression is not necessarily altered in the sample, but rather expression of genes in those genes’ pathways are elevated versus reference.

The divergence topological method identifies genes in the interactome knowledge base that are implicated by the submitted gene list as having significantly enrichment for divergence in the shortest path analysis between all the genes in the submitted list. This implies that an inhibition of that gene may affect many of the downstream gene expression values.

The third topological method focuses on genes in the interactome that have direct interaction with transcription factors and have significant enrichment for the shortest path analysis between all the submitted genes in the list. These genes, if they are the target of a drug, can have significant effect downstream on the over expressed genes in the submitted list.

In this study, all three methods are utilized; however, the third method referred to as the network drug target method has been the main focus. The other two methods have been deemphasized by imposing very large limits on the negative log p-values of enrichment.

The output from all three methods is a list of Entrez genes with associated p-values of enrichment. These p-values must exceed a limit before being reported and the score is derived from the negative log p-value reported for each gene. The genes are used to identify drugs from the first index of drug knowledge base containing known gene expression inhibitions by drugs.

## Drug Response Signatures (Connectivity Map or CMAP)

The method was developed by the Broad Institute and uses publically available data sets. The original paper by Lamb et al[4] showed that it was possible to identify patterns of effects that drug produced on cell lines by examining pre- and post-treatment gene expression profiles. The method is independently implemented and has been validated against the original results produced by the available online tool. This method uses probes with z-scores greater than or equal 1.5 limited to the top 500 probes and the z-scores less than or equal -1.5 limited to bottom 500 probes. The score is calculated using Kolmogorov-Smirnov statistics with p-values estimated using permutation testing. The number of permutations is 50,000 and only those patterns that match with a p-value less than 0.05 are reported. The number of drugs in the CMAP training library is extensive but only those drugs that were supported by other criteria are included in summary calculations and reported (see Additional File 2).

# Drug Sensitivity Signatures (PGSEA)

The PGSEA algorithm uses the published NCI-60 cell line sensitivity to drugs to produce a limited set of gene over expression signatures which that drug sensitivity as indicated by decreased IC-50 values for the drug. A one sample t-test is applied to evaluate the potential sensitivity to a signature. The method uses a list of the top positive z-scores mapped to Entrez gene id. Only patterns that match in a positive value, representing increased expression in sensitivity-indicating signatures, are reported.

## Method Variance

The limits for list size caps and z-score thresholds are all subject to experimental design and can be adjusted for individual purposes. The limits and size caps as documented in this description form the default values for the PMED reports that are produced. There are additional limits placed on the probes for minimum expression values and reported present condition prior to calculation of the z-scores and only those probes that exceed the limits are included in the analysis.

Post-analysis of the topological methods and the drug target expression genes for experimental drugs can also be performed. Here, post-analysis for GeneGo pathway alterations, irrespective of pharmacological relevance, identified significant up-regulation in DNA damage response pathways, as shown in detail in Figure 5.

1. Overington JP, Al-Lazikani B, Hopkins AL: **How many drug targets are there?** *Nat Rev Drug Discov* 2006, **5:**993-996.

2. Dezso Z, Nikolsky Y, Nikolskaya T, Miller J, Cherba D, Webb C, Bugrim A: **Identifying disease-specific genes based on their topological significance in protein networks.** *BMC Syst Biol* 2009, **3:**36.

3. Furge KA, Chen J, Koeman J, Swiatek P, Dykema K, Lucin K, Kahnoski R, Yang XJ, Teh BT: **Detection of DNA copy number changes and oncogenic signaling abnormalities from gene expression data reveals MYC activation in high-grade papillary renal cell carcinoma.** *Cancer Res* 2007, **67:**3171-3176.

4. Lamb J, Crawford ED, Peck D, Modell JW, Blat IC, Wrobel MJ, Lerner J, Brunet JP, Subramanian A, Ross KN, et al: **The Connectivity Map: using gene-expression signatures to connect small molecules, genes, and disease.** *Science* 2006, **313:**1929-1935.

5. Knox C, Law V, Jewison T, Liu P, Ly S, Frolkis A, Pon A, Banco K, Mak C, Neveu V, et al: **DrugBank 3.0: a comprehensive resource for 'omics' research on drugs.** *Nucleic Acids Res* 2011, **39:**D1035-1041.

6. Von Hoff DD, Stephenson JJ, Jr., Rosen P, Loesch DM, Borad MJ, Anthony S, Jameson G, Brown S, Cantafio N, Richards DA, et al: **Pilot study using molecular profiling of patients' tumors to find potential targets and select treatments for their refractory cancers.** *J Clin Oncol* 2010, **28:**4877-4883.
