# Supplementary material for: Molecular-guided therapy predictions reveal drug resistance phenotypes and treatment alternatives in malignant peripheral nerve sheath tumors
Source: J Transl Med. 2013 Sep 17;11:213. doi: 10.1186/1479-5876-11-213 (PMC3848568; doi:10.1186/1479-5876-11-213)
Supplement: Additional file 2 — Detailed summary reports of molecular-guided therapy predictions. A summary spreadsheet is provided detailing the summary results for the analyzed MTTB neurofibroma samples (A-G) and MPNSTs from the public data set (A’-G’). Ranked therapeutics and corresponding scores as indicated for each sample (A) are based upon intermediate results from B) drug target expression, C) network topology and target activity, D) parametric gene set enrichment analysis (PGSEA), E) connectivity map (CMAP) analysis, F) biomarker based rules (resistant) analysis, and G) biomarker rules (sensitive) analysis. [file 1479-5876-11-213-S2.pdf]

A. Therapy drug score summary by sample: Neurofibromas

(-) indicates no score

Drug	NF_MS 135T	NF_MS 142T	NF_MS 153T	NF_MS 156T	NF_MS 34T	NF_MS 37T	NF_MS 90T
amiloride	16.15	-	8.28	-	-	10.00	-
amiodarone	-	-	4.91	-	-	-	-
bevacizumab	-	-	-	2.49	-	-	-
biperiden	10.00	-	-	-	3.36	-	10.00
bortezomib	11.75	-	-	-	-	-	-
brompheniramine	-	-	-	2.58	-	-	-
caffeine	-	-	-	2.49	-	-	-
carbinoxamine	10.00	-	-	2.58	-	-	10.00
cetirizine	-	-	-	2.58	-	-	-
cetuximab	-	-	4.84	-	-	-	5.05
cimetidine	-	-	-	2.49	-	-	-
clemastine	-	-	-	2.58	-	-	-
clofarabine	-	-	-	3.22	-	5.70	6.99
clozapine	16.07	3.40	-	-	-	-	10.00
cyclobenzaprine	-	3.40	-	-	-	-	-
cyproheptadine	-	3.40	-	2.58	-	-	-
dasatinib	32.08	4.88	10.00	-	-	15.49	5.04
daunorubicin	-	6.13	-	-	-	6.30	-
doxycycline	-	-	-	-	-	9.70	-
dutasteride	-	-	-	-	-	5.42	-
erlotinib	-	-	4.84	-	-	-	5.05
finasteride	11.90	-	-	-	2.64	9.12	-
flavoxate	10.00	-	-	-	-	-	10.00
fludarabine	-	-	-	3.22	-	5.70	6.99
fludrocortisone	-	-	-	-	2.57	-	-
fluticasone	-	10.00	-	-	-	-	-
fulvestrant	-	3.64	-	-	-	-	-
gefitinib	-	-	4.84	-	-	-	5.05
gentamicin	-	-	-	-	2.52	-	-
imatinib	14.42	-	10.00	-	-	12.49	-
lapatinib	-	-	4.84	-	-	-	5.05
lenalidomide	-	-	-	2.49	-	-	-
medroxyprogesterone	-	10.00	-	-	-	-	-
megestrol	-	-	-	-	-	-	4.37
nilotinib	14.42	-	10.00	-	-	14.98	-
octreotide	14.49	-	-	-	-	-	-
pamidronate	-	-	10.00	-	-	-	-
panitumumab	-	-	-	-	-	-	5.05
pazopanib	18.37	-	10.00	-	-	14.42	-
pravastatin	-	9.35	-	-	-	-	-
raloxifene	-	3.64	-	-	-	-	4.37
romidepsin	-	-	-	-	-	4.90	-
sorafenib	23.84	10.00	10.00	5.28	-	14.42	-
sunitinib	18.37	10.00	10.00	5.28	-	14.42	-
tamoxifen	-	9.77	-	-	-	-	-
thioguanine	-	-	5.25	-	-	-	-
trazodone	-	6.26	-	2.58	-	-	-
vandetanib	-	-	-	-	-	-	5.05
vorinostat	17.47	4.82	-	2.67	-	8.04	-
zoledronic acid	-	-	10.00	-	-	-	-

A'. Therapy drug score summary by sample: MPNSTs

Drug	mpnst 02_2	mpnst _94_3	mpnst 95_3b	mpnst _as10	mpnst _as13	mpnst _as15	mpnst _as37	mpnst _as42	mpnst _as45	mpnst 96_2	mpnst 97_6
abciximab	-	-	3.66	-	-	-	4.42	-	3.79	-	-
acarbose	-	-	-	-	-	-	3.05	-	-	-	-
acetylsalicylic acid	8.76	-	-	-	-	-	-	-	-	3.51	-
alfuzosin	8.2	-	-	-	-	-	-	-	1.65	-	-
amiodarone	-	-	-	-	3.33	-	-	-	-	-	-
amitriptyline	-	-	-	1.65	-	-	1.35	-	-	-	-
anakinra	-	4.73	-	-	-	-	-	-	-	-	-
balsalazide	8.76	-	-	-	-	-	-	-	-	3.51	-
betaxolol	-	-	3.66	-	-	-	-	-	-	6.83	-
bevacizumab	-	-	3.66	2.93	-	-	-	3.35	-	-	-
bortezomib	-	-	-	-	-	-	-	-	3.98	4.32	-
brinzolamide	-	-	-	-	-	-	1.43	-	-	-	-
bromfenac	8.76	-	-	-	-	-	-	-	-	3.51	-
brompheniramine	-	4.67	-	-	10	-	-	-	-	-	-
bumetanide	-	-	-	-	-	-	2.04	-	-	-	-
caffeine	-	-	-	2.85	6.06	2.99	-	-	-	12.3	-
carbinoxamine	-	4.67	-	-	10	-	-	-	-	-	-
carmustine	-	4.95	2.1	-	-	-	-	-	2.56	5.87	3.25
celecoxib	8.76	-	3.48	-	3.94	-	-	-	-	3.51	-
cetirizine	-	4.67	-	-	10	-	-	-	-	-	-
cetuximab	**5.27**	-	-	-	-	-	**4.93**	-	-	-	-
chlorpromazine	-	-	-	-	-	-	-	-	-	13.8	-
cimetidine	-	-	3.66	2.93	-	-	-	3.35	-	-	-
ciprofloxacin	10	4.46	-	10	10	9.96	4.53	-	10	4.47	-
cisplatin	-	3.33	3.07	-	-	-	-	-	-	-	2.3
clemastine	-	4.67	-	-	10	-	-	-	-	-	-
clozapine	-	-	-	-	-	2.42	-	-	-	3.84	12.1
colchicine	-	3.17	-	-	-	-	-	-	-	-	-
cyclobenzaprine	-	-	-	-	-	-	-	-	-	-	10
cyclosporin	-	-	4.92	-	-	-	-	-	1.35	8.01	2.92
cyproheptadine	-	4.67	-	-	10	-	-	-	-	-	10
dasatinib	9.16	-	4.76	3.05	-	4.31	10.4	-	8.73	12.7	3.64
daunorubicin	11.4	6.7	-	10	10	9.96	4.53	-	12.2	4.47	-
diflunisal	8.76	-	-	-	-	-	-	-	-	3.51	-
disulfiram	-	-	-	2.1	-	2.13	-	-	1.55	-	-
docetaxel	-	3.17	-	-	-	-	-	-	-	-	-
dorzolamide	-	-	-	-	-	-	-	2.68	-	-	-
doxazosin	-	-	-	-	-	-	-	-	1.65	-	-
doxorubicin	-	-	-	11.7	11.5	11.5	6.38	-	14.3	7.35	-
doxycycline	8.52	-	-	14.1	-	3.28	20	22.1	12.9	-	-
droperidol	-	-	-	-	-	-	-	-	-	3.84	-
drotrecogin alfa	-	-	**3.12**	**5.34**	-	**2.42**	**4.6**	-	**5.03**	-	**3.05**
epirubicin	10	4.46	-	10	10	9.96	4.53	-	12.2	4.47	-
eribulin	-	3.17	-	-	-	-	-	-	-	-	-
erlotinib	-	-	-	-	-	-	4.93	-	-	-	-
estradiol	-	-	-	-	-	-	2.19	-	-	-	-
ethosuximide	-	-	-	3.3	-	-	3.27	-	4.52	-	-
etodolac	8.76	-	-	-	-	-	-	-	-	3.51	-
etoposide	11.7	4.46	1.79	11.4	12.2	11.7	4.53	-	14.2	5.96	-
everolimus	9.59	-	-	-	-	-	-	-	-	-	4.17

fenoprofen	8.76	-	-	-	-	-	-	-	-	3.51	-
finasteride	-	-	-	-	-	-	2.65	-	-	-	-
flavoxate	10	10	-	-	-	-	-	-	-	-	-
fludrocortisone	-	-	-	-	-	-	1.62	-	-	-	-
fluphenazine	-	-	1.47	2.01	-	1.92	-	-	-	-	-
flutamide	-	-	1.77	11.8	6.61	11.8	10	-	7.27	-	-
fluticasone	-	-	-	-	-	-	4.63	-	-	-	-
fulvestrant	-	-	-	-	-	-	3.93	-	-	-	-
gefitinib	9.18	-	3.66	-	-	-	4.93	-	-	4.85	-
guanethidine	8.2	-	9.63	-	-	1.96	1.77	-	1.65	4.84	10
hydrochlorothiazide	-	1.88	-	-	-	-	-	-	-	-	-
ibuprofen	8.76	-	-	-	-	-	-	-	-	3.51	-
iloprost	**-**	**-**	**-**	**-**	**-**	**-**	**1.58**	**-**	**-**	**-**	**-**
imatinib	9.16	-	-	8.4	-	4.49	4.12	4.95	-	8.32	4.99
ixabepilone	-	3.17	-	-	-	-	-	-	-	-	-
ketorolac	8.76	-	-	-	-	-	-	-	-	3.51	-
lansoprazole	-	-	-	-	1.43	-	-	-	-	-	-
lapatinib	9.18	-	3.66	-	-	-	4.93	-	-	4.85	-
leflunomide	8.76	-	-	3.05	-	-	-	-	-	3.51	-
lenalidomide	12.7	-	3.66	2.93	-	-	-	3.35	-	3.51	-
lomustine	6.58	3.61	2.81	4.22	2.35	4.7	1.43	-	3.8	5.71	4.25
megestrol	6.81	4.87	2.83	-	-	-	-	-	-	-	1.54
meloxicam	8.76	-	-	-	-	-	-	-	-	3.51	-
melphalan	-	4.73	-	-	-	-	-	-	-	-	5.21
methotrexate	-	-	-	6.04	-	8.04	-	-	-	-	-
minocycline	-	-	-	-	-	-	10	10	7.45	-	-
mitoxantrone	10	4.46	-	10	10	9.96	4.53	-	10	4.47	-
nabumetone	8.76	-	-	-	-	-	-	-	-	-	-
naproxen	-	-	-	-	-	-	1.34	-	-	-	-
nilotinib	9.16	-	-	3.05	-	-	4.12	-	-	8.32	-
octreotide	-	6.15	-	-	-	-	-	22.2	22	-	12.6
oxaliplatin	9.59	-	-	-	-	-	-	-	-	7.54	-
paclitaxel	-	3.17	-	-	-	-	-	-	-	3.75	-
paclitaxel albuminbound	**-**	**3.17**	**-**	**-**	**-**	**-**	**-**	**-**	**-**	**-**	**-**
pamidronate	-	-	-	-	2.64	-	-	-	-	-	-
panitumumab	-	-	-	-	-	-	4.93	-	-	-	-
pazopanib	-	-	3.66	20.6	-	10	4.12	5.63	-	3.87	-
pemetrexed	6.2	-	-	6.04	-	8.04	-	-	-	-	-
phentolamine	-	-	2.6	-	-	1.96	-	-	-	-	-
piperazine	-	1.4	-	-	-	1.39	-	-	-	-	-
plerixafor	-	-	-	-	-	3.26	3.45	-	-	-	4.89
pralatrexate	-	-	-	6.04	-	8.04	-	-	-	-	-
pravastatin	-	14.4	-	-	-	-	13.6	13.7	10.8	-	8.8
prochlorperazine	-	-	-	-	-	-	-	-	-	3.84	-
raloxifene	6.81	4.87	2.83	-	-	-	3.93	-	-	-	1.54
romidepsin	5.71	-	-	-	-	-	-	-	4.13	3.68	-
sildenafil	7.52	10	-	-	-	-	-	-	-	-	-
simvastatin	5.45	2.64	-	3.8	-	-	-	-	3.52	4.08	-
sirolimus	15.1	-	4.7	-	-	-	-	-	-	-	4.17
sorafenib	-	2.78	3.66	20.6	5.09	10	7.29	8.97	4.26	3.87	-
sunitinib	-	-	3.66	20.6	1.79	12.8	7.29	5.63	3.15	3.87	-
tacrolimus	-	-	4.92	-	-	-	-	-	1.35	8.01	2.92
tadalafil	7.52	10	-	-	-	-	-	-	-	-	-
tamoxifen	6.81	4.87	2.83	-	-	-	3.93	-	-	-	1.54

temsirolimus	**9.59**	-	-	-	-	-	-	-	-	-	**4.17**
teniposide	20	8.92	-	20	20	19.9	9.07	-	22.2	8.94	-
thalidomide	7.91	4.73	3.66	2.93	-	-	-	3.35	-	4.1	9.29
thioguanine	5.46	-	6.49	-	-	-	-	2.98	5.03	-	2.22
thioridazine	-	-	2.49	4.7	-	4.7	-	-	1.65	10.4	10
thiotepa	-	1.4	-	-	-	1.39	-	-	-	-	-
tolmetin	8.76	-	-	-	-	-	-	-	-	-	-
topotecan	8.61	-	-	-	-	-	-	-	-	5.91	-
toremifene	6.81	4.87	2.83	-	-	-	-	-	-	-	1.54
trastuzumab	-	-	3.66	-	-	-	-	-	-	4.85	-
trazodone	-	4.67	2.6	-	10	4.05	1.51	-	1.65	-	10
valproic acid	-	-	-	-	-	-	-	-	-	3.71	-
valrubicin	10	4.46	-	10	10	9.96	4.53	-	12.2	4.47	-
vandetanib	-	-	3.66	17.6	-	10	4.93	5.63	-	-	-
vardenafil	7.52	10	-	-	-	-	-	-	-	-	-
vinblastine	-	3.17	-	-	-	-	-	2.9	-	-	-
vinorelbine	-	3.17	-	-	-	-	-	-	-	-	-
vorinostat	32.7	-	-	14.6	8.09	16.8	11.4	3.11	17.2	15.5	-
yohimbine	-	-	9.63	-	-	1.96	1.77	-	-	-	10
zoledronic acid	-	-	-	-	2.64	-	-	-	-	-	-

B. Drug Target Expression

Drug_Drug Taget Gene	135T	142T	153T	156T	34T	37T	90T
amiloride_SCNN1A	97.57	-	-	-	-	-	-
amiloride_SCNN1B	-	-	8.28	-	-	-	-
amiloride_SCNN1G	6.15	-	-	-	-	11.42	-
amiodarone_KCNJ8	5.64	-	4.91	-	-	-	-
atorvastatin_HMGCR	-	2.97	-	-	-	-	-
azacitidine_DNMT1	2.93	-	-	-	-	-	-
biperiden_CHRM1	22.83	-	-	-	-	-	12.10
bortezomib_AKT1	-	-	-	-	-	-	-
brompheniramine_HRH1	-	-	-	2.58	-	-	-
caffeine_ADORA1	3.37	-	-	-	-	-	-
carbinoxamine_CHRM1	22.83	-	-	-	-	-	12.10
carbinoxamine_HRH1	-	-	-	2.58	-	-	-
cetirizine_HRH1	-	-	-	2.58	-	-	-
clemastine_HRH1	-	-	-	2.58	-	-	-
clofarabine_POLA1	-	-	-	3.22	-	5.70	6.99
clofarabine_RRM1	-	-	-	-	-	-	-
clozapine_CHRM1	22.83	-	-	-	-	-	12.10
clozapine_HTR2A	-	3.40	-	-	-	-	-
colchicine_TUBB2A	-	-	3.62	-	-	-	-
cyclobenzaprine_HTR2A	-	3.40	-	-	-	-	-
cyproheptadine_HRH1	-	-	-	2.58	-	-	-
cyproheptadine_HTR2A	-	3.40	-	-	-	-	-
dasatinib_EPHA2	4.04	4.88	-	-	-	-	-
dasatinib_KIT	56.20	-	29.36	-	-	39.22	-
daunorubicin_ABCB1	3.48	6.13	-	-	-	-	-
decitabine_DNMT1	2.93	-	-	-	-	-	-
denileukin diftitox_IL2RB	-	-	2.77	-	-	-	-
dorzolamide_CA2	3.83	-	-	-	-	-	-
doxorubicin_ABCB1	3.48	6.13	-	-	-	-	-
doxycycline_MMP3	-	2.97	-	-	-	9.70	-

B'. Drug Target Expression

Drug_Drug Taget Gene	mpnst 02_2	mpnst _94_3	mpnst 95_3b	mpnst _as10	mpnst _as13	mpnst _as15	mpnst _as37	mpnst _as42	mpnst _as45	mpnst 96_2	mpnst 97_6
acarbose_GAA	-	-	-	-	-	-	3.05	-	-	-	-
amiodarone_KCNJ8	-	-	-	-	3.33	-	-	-	-	-	-
anakinra_IL1R1	-	4.73	-	-	-	-	-	-	-	-	-
azacitidine_DNMT1	3.33	-	-	-	-	-	-	-	-	-	-
brompheniramine_HRH1	-	4.67	-	-	11.2	-	-	-	-	-	-
caffeine_ADORA2B	-	-	-	-	2.62	-	-	-	-	82.1	-
carbinoxamine_HRH1	-	4.67	-	-	11.2	-	-	-	-	-	-
celecoxib_PDPK1	-	-	3.48	-	-	-	-	-	-	-	-
cetirizine_HRH1	-	4.67	-	-	11.2	-	-	-	-	-	-
chlorpromazine_HTR7	-	-	-	-	-	-	-	-	-	100	-
ciprofloxacin_TOP2A	22.3	4.46	-	15.1	12.3	9.96	4.53	-	20.2	4.47	-
clemastine_HRH1	-	4.67	-	-	11.2	-	-	-	-	-	-
clofarabine_RRM1	4.71	-	-	-	-	-	-	-	-	-	-
clozapine_HTR2A	-	-	-	-	-	-	-	-	-	-	12.7
colchicine_TUBB	3.38	3.17	-	-	-	-	-	-	-	-	-
cyclobenzaprine_HTR2A	-	-	-	-	-	-	-	-	-	-	12.7
cyclosporin_PPP3CB	-	-	4.92	-	-	-	-	-	-	2.98	2.92
cyproheptadine_HRH1	-	4.67	-	-	11.2	-	-	-	-	-	-
cyproheptadine_HTR2A	-	-	-	-	-	-	-	-	-	-	12.7
daunorubicin_TOP2A	22.3	4.46	-	15.1	12.3	9.96	4.53	-	20.2	4.47	-
decitabine_DNMT1	**3.33**	-	-	-	-	-	-	-	-	-	-
docetaxel_TUBB	3.38	3.17	-	-	-	-	-	-	-	-	-
dorzolamide_CA2	-	-	-	-	-	-	-	2.68	-	-	-
doxorubicin_TOP2A	22.3	4.46	-	15.1	12.3	9.96	4.53	-	20.2	4.47	-
doxycycline_MMP13	-	-	-	8.23	-	-	82.7	100	-	-	-
doxycycline_MMP9	-	-	-	-	-	-	100	100	7.45	-	-
drotrecogin alfa_SERPINE1	-	-	3.12	-	-	-	-	-	-	-	3.05
epirubicin_TOP2A	22.3	4.46	-	15.1	12.3	9.96	4.53	-	20.2	4.47	-
eribulin_TUBB	3.38	3.17	-	-	-	-	-	-	-	-	-
ethosuximide_CACNA1G	-	-	-	3.3	-	-	3.27	-	4.52	-	-

drotrecogin alfa_SERPINE1	-	-	-	-	-	-	-
dutasteride_SRD5A1	4.71	-	-	-	-	5.42	-
eribulin_TUBB2A	-	-	3.62	-	-	-	-
finasteride_SRD5A1	4.71	-	-	-	-	5.42	-
flavoxate_CHRM1	22.83	-	-	-	-	-	12.10
fludarabine_POLA1	-	-	-	3.22	-	5.70	6.99
fluticasone_PGR	-	13.87	-	-	-	-	-
fulvestrant_ESR2	-	3.64	-	-	-	-	-
imatinib_KIT	56.20	-	29.36	-	-	39.22	-
irinotecan_TOP1	-	-	4.36	-	-	-	-
ixabepilone_TUBB2A	-	-	3.62	-	-	-	-
leflunomide_DHODH	-	2.87	-	-	-	-	-
lovastatin_HMGCR	**-**	**2.97**	**-**	**-**	**-**	**-**	**-**
methotrexate_DHFR	-	-	3.68	-	-	-	-
nilotinib_KIT	56.20	-	29.36	-	-	39.22	-
octreotide_SSTR1	6.73	-	-	-	-	-	-
octreotide_SSTR2	4.75	-	-	-	-	-	-
paclitaxel albumin-bound_TUBB2A	-	-	3.62	-	-	-	-
paclitaxel_TUBB2A	-	-	3.62	-	-	-	-
pamidronate_FDPS	12.52	-	10.08	-	-	-	-
pazopanib_KIT	56.20	-	29.36	-	-	39.22	-
pemetrexed_DHFR	-	-	3.68	-	-	-	-
pralatrexate_DHFR	-	-	3.68	-	-	-	-
pravastatin_HMGCR	-	2.97	-	-	-	-	-
raloxifene_ESR2	-	3.64	-	-	-	-	-
simvastatin_HMGCR	-	2.97	-	-	-	-	-
sorafenib_FLT3	-	71.29	-	5.28	-	-	-
sorafenib_KIT	56.20	-	29.36	-	-	39.22	-
sunitinib_FLT3	-	71.29	-	5.28	-	-	-
sunitinib_KIT	56.20	-	29.36	-	-	39.22	-
tamoxifen_ABCB1	3.48	6.13	-	-	-	-	-
tamoxifen_ESR2	-	3.64	-	-	-	-	-
thioguanine_IMPDH1	-	-	5.25	-	-	-	-
thioridazine_HTR2A	-	3.40	-	-	-	-	-
topotecan_TOP1	-	-	4.36	-	-	-	-
trazodone_HRH1	-	-	-	2.58	-	-	-
trazodone_HTR2A	-	3.40	-	-	-	-	-
vinblastine_TUBB2A	-	-	3.62	-	-	-	-
vinorelbine_TUBB2A	-	-	3.62	-	-	-	-
vorinostat_HDAC3	-	-	-	2.67	-	-	-
zoledronic acid_FDPS	12.52	-	10.08	-	-	-	-

etoposide_TOP2A	22.3	4.46	-	15.1	12.3	9.96	4.53	-	20.2	4.47	-
flavoxate_CHRM2	18.3	100	-	-	-	-	-	-	-	-	-
fludarabine_RRM1	4.71	-	-	-	-	-	-	-	-	-	-
flutamide_PEG10	-	-	-	100	6.61	100	32.8	-	7.27	-	-
guanethidine_ADRA2C	-	-	-	-	-	-	-	-	-	-	13.8
hydroxyurea_RRM1	4.71	-	-	-	-	-	-	-	-	-	-
imatinib_PDGFC	-	-	-	3.92	-	4.49	-	3.64	-	-	2.66
irinotecan_TOP1	4.3	-	-	-	-	-	-	-	-	2.95	-
ixabepilone_TUBB	3.38	3.17	-	-	-	-	-	-	-	-	-
melphalan_MMP2	-	4.73	-	-	-	-	-	-	-	-	5.21
methotrexate_DHFR	-	-	-	6.04	-	8.04	-	-	-	-	-
minocycline_MMP9	**-**	**-**	**-**	**-**	**-**	**-**	**100**	**100**	**7.45**	**-**	**-**
mitoxantrone_TOP2A	22.3	4.46	-	15.1	12.3	9.96	4.53	-	20.2	4.47	-
octreotide_SSTR1	3.17	6.15	-	-	-	-	-	-	-	-	22.8
octreotide_SSTR2	-	-	-	-	-	-	-	23.7	12.5	-	-
paclitaxel albuminbound_TUBB	3.38	3.17	-	-	-	-	-	-	-	-	-
paclitaxel_TUBB	3.38	3.17	-	-	-	-	-	-	-	-	-
pamidronate_FDPS	-	-	-	-	2.64	-	-	-	-	-	-
pazopanib_FLT1	-	-	-	-	-	-	-	5.63	-	-	-
pazopanib_KDR	-	-	-	32	-	28.7	-	-	-	-	-
pemetrexed_ATIC	6.2	-	-	-	-	-	-	-	-	-	-
pemetrexed_DHFR	-	-	-	6.04	-	8.04	-	-	-	-	-
pralatrexate_DHFR	-	-	-	6.04	-	8.04	-	-	-	-	-
pravastatin_MMP14	-	3.44	-	-	-	-	-	-	-	-	-
pravastatin_MMP2	-	4.73	-	-	-	-	-	-	-	-	5.21
pravastatin_MMP9	-	-	-	-	-	-	100	100	7.45	-	-
sildenafil_PDE5A	7.52	13.1	-	-	-	-	-	-	-	-	-
sorafenib_BRAF	-	-	-	-	3.3	-	-	3.35	-	-	-
sorafenib_FLT1	-	-	-	-	-	-	-	5.63	-	-	-
sorafenib_KDR	-	-	-	32	-	28.7	-	-	-	-	-
sorafenib_RAF1	3.08	2.78	-	-	-	-	-	-	-	-	-
sunitinib_FLT1	-	-	-	-	-	-	-	5.63	-	-	-
sunitinib_KDR	**-**	**-**	**-**	**32**	**-**	**28.7**	**-**	**-**	**-**	**-**	**-**
tacrolimus_PPP3CB	-	-	4.92	-	-	-	-	-	-	2.98	2.92
tadalafil_PDE5A	7.52	13.1	-	-	-	-	-	-	-	-	-
teniposide_TOP2A	22.3	4.46	-	15.1	12.3	9.96	4.53	-	20.2	4.47	-
thalidomide_FGF2	-	-	-	-	-	-	-	-	-	-	4.08
thalidomide_MMP2	-	4.73	-	-	-	-	-	-	-	-	5.21
thioguanine_HPRT1	3.92	-	3.59	-	-	-	-	2.98	-	-	-
thioguanine_PPAT	-	-	-	-	-	-	-	-	3.65	-	-
thioridazine_HTR2A	-	-	-	-	-	-	-	-	-	-	12.7
topotecan_TOP1	4.3	-	-	-	-	-	-	-	-	2.95	-
trazodone_HRH1	-	4.67	-	-	11.2	-	-	-	-	-	-
trazodone_HTR2A	-	-	-	-	-	-	-	-	-	-	12.7
valrubicin_TOP2A	22.3	4.46	-	15.1	12.3	9.96	4.53	-	20.2	4.47	-
vandetanib_FLT1	-	-	-	-	-	-	-	5.63	-	-	-
vandetanib_KDR	-	-	-	32	-	28.7	-	-	-	-	-
vardenafil_PDE5A	7.52	13.1	-	-	-	-	-	-	-	-	-
vinblastine_TUBB	3.38	3.17	-	-	-	-	-	-	-	-	-
vinorelbine_TUBB	3.38	3.17	-	-	-	-	-	-	-	-	-
vorinostat_HDAC2	5.89	-	-	7.87	5.38	10	-	-	-	-	-
yohimbine_ADRA2C	-	-	-	-	-	-	-	-	-	-	13.8
zoledronic acid_FDPS	-	-	-	-	2.64	-	-	-	-	-	-

C. Network Topology & Target Activity (GeneGo)

Drug_Network Target	135T	142T	153T	156T	34T	37T	90T
abciximab_ITGB3	4.19	-	-	-	-	3.70	-
betaxolol_ADRB2	2.97	-	-	-	-	3.07	-
bevacizumab_VEGFA	-	-	-	2.49	-	-	-
bicalutamide_AR	7.19	-	3.48	-	-	3.70	-
bortezomib_AKT1	6.29	-	-	-	-	4.61	-
bortezomib_NFKB1	5.46	-	-	-	-	-	-
caffeine_ADORA2A	-	-	-	2.49	-	-	-
carbidopa_DDC	5.42	-	-	-	-	-	-
celecoxib_PDPK1	-	-	3.05	-	-	-	-
cetuximab_EGFR	-	-	4.84	-	-	-	5.05
chlorpromazine_DRD2	2.38	-	-	-	-	-	-
cimetidine_VEGFA	-	-	-	2.49	-	-	-
ciprofloxacin_TOP2A	-	-	-	-	-	4.23	-
clozapine_DRD2	2.38	-	-	-	-	-	-
clozapine_DRD3	3.69	-	-	-	-	-	-
dasatinib_ABL1	-	-	-	-	-	2.49	-
dasatinib_EPHA2	3.20	-	-	-	-	3.00	-
dasatinib_KIT	4.42	-	-	-	-	-	-
dasatinib_LCK	4.88	-	-	-	-	-	-
dasatinib_SRC	5.54	-	-	-	-	-	5.04
daunorubicin_TOP2A	-	-	-	-	-	4.23	-
denileukin diftitox_IL2RG	-	-	-	-	-	2.39	-
doxorubicin_TOP2A	-	-	-	-	-	4.23	-
doxycycline_IL1A	-	-	-	-	-	-	3.11
doxycycline_MMP9	4.18	-	-	-	-	-	-
droperidol_DRD2	2.38	-	-	-	-	-	-
epirubicin_TOP2A	-	-	-	-	-	4.23	-
erlotinib_EGFR	-	-	4.84	-	-	-	5.05
etoposide_TOP2A	-	-	-	-	-	4.23	-
finasteride_AR	7.19	-	3.48	-	-	3.70	-
flutamide_AR	7.19	-	3.48	-	-	3.70	-
fulvestrant_AR	7.19	-	3.48	-	-	3.70	-
gefitinib_EGFR	-	-	4.84	-	-	-	5.05
guanethidine_ADRA2A	-	2.86	-	-	-	-	-
imatinib_ABL1	-	-	-	-	-	2.49	-
imatinib_KIT	4.42	-	-	-	-	-	-
irinotecan_TOP1	-	-	-	-	-	-	-
lapatinib_EGFR	-	-	4.84	-	-	-	5.05
lenalidomide_VEGFA	-	-	-	2.49	-	-	-
megestrol_ESR1	4.53	-	-	-	-	4.39	4.37
minocycline_MMP9	4.18	-	-	-	-	-	-
mitoxantrone_TOP2A	-	-	-	-	-	4.23	-
nilotinib_ABL1	-	-	-	-	-	2.49	-
nilotinib_BCR	-	-	-	-	-	2.49	-
nilotinib_KIT	4.42	-	-	-	-	-	-
nilutamide_AR	7.19	-	3.48	-	-	3.70	-
octreotide_SSTR2	-	-	-	-	-	-	-
octreotide_SSTR5	3.01	-	-	-	-	-	-
oxaliplatin_BCL2L1	3.12	-	-	-	-	-	-
panitumumab_EGFR	-	-	4.84	-	-	-	5.05
pazopanib_KDR	3.95	-	-	-	-	4.42	-

C'. Network Topology & Target Activity (GeneGo)

Drug_Network Target	mpnst 02_2	mpnst _94_3	mpnst 95_3b	mpnst _as10	mpnst _as13	mpnst _as15	mpnst _as37	mpnst _as42	mpnst _as45	mpnst 96_2	mpnst 97_6
abciximab_ITGB3	5.17	-	3.66	-	-	-	4.42	-	3.79	-	-
acetylsalicylic acid_PTGS2	8.76	-	-	-	-	-	-	-	-	3.51	-
adalimumab_TNF	3.98	-	-	-	-	-	-	-	-	-	-
alfuzosin_ADRA1A	4.45	-	-	-	-	-	-	-	1.65	1.85	-
alfuzosin_ADRA1B	3.76	-	-	-	-	-	-	-	-	-	-
balsalazide_PTGS2	8.76	-	-	-	-	-	-	-	-	3.51	-
betaxolol_ADRB1	-	-	-	-	-	-	-	-	-	3.38	-
betaxolol_ADRB2	-	-	3.66	-	-	-	-	-	-	3.45	-
bevacizumab_VEGFA	-	-	3.66	2.93	-	-	-	3.35	-	-	-
bisoprolol_ADRB1	-	-	-	-	-	-	-	-	-	3.38	-
bortezomib_AKT1	-	-	-	-	-	-	-	-	3.98	4.32	-
bortezomib_NFKB1	3.93	-	-	-	-	-	-	-	-	-	-
bromfenac_PTGS2	8.76	-	-	-	-	-	-	-	-	3.51	-
caffeine_ADORA1	-	-	-	1.47	1.75	1.37	-	-	-	-	-
caffeine_ADORA2B	2.36	-	-	1.37	1.69	1.62	-	-	-	2.29	-
celecoxib_PDPK1	-	-	-	-	3.94	-	-	-	-	-	-
celecoxib_PTGS2	8.76	-	-	-	-	-	-	-	-	3.51	-
cetuximab_EGFR	5.27	-	-	-	-	-	4.93	-	-	-	-
chlorpromazine_DRD2	-	-	-	-	-	-	-	-	-	3.84	-
cimetidine_VEGFA	-	-	3.66	2.93	-	-	-	3.35	-	-	-
clozapine_DRD2	**-**	**-**	**-**	**-**	**-**	**-**	**-**	**-**	**-**	**3.84**	**-**
clozapine_DRD3	-	-	-	-	-	2.42	-	-	-	-	2.07
cyclosporin_PPP3CA	-	-	-	-	-	-	-	-	1.35	-	-
cyclosporin_PPP3R1	2.18	-	-	-	-	-	-	-	-	2.51	-
cyclosporin_PPP3R2	2.18	-	-	-	-	-	-	-	-	2.51	-
dasatinib_ABL1	9.16	-	-	-	-	-	-	-	-	4.45	-
dasatinib_FYN	-	-	-	-	-	-	-	-	-	4.38	-
dasatinib_KIT	-	-	-	-	-	-	4.12	-	-	3.87	-
dasatinib_LCK	-	-	-	-	-	-	-	-	3.54	-	3.64
dasatinib_PDGFRB	-	-	-	3.05	-	-	-	-	-	-	-
dasatinib_SRC	-	-	4.76	-	-	4.31	6.3	-	5.19	-	-
daunorubicin_TOP2B	-	-	-	-	-	-	-	-	2.19	-	-
denileukin diftitox_IL2RG	2.02	-	-	-	-	-	-	-	-	-	-
diflunisal_PTGS2	8.76	-	-	-	-	-	-	-	-	3.51	-
doxazosin_ADRA1A	4.45	-	-	-	-	-	-	-	1.65	1.85	-
doxorubicin_TOP2B	-	-	-	-	-	-	-	-	2.19	-	-
doxycycline_IL1A	-	-	-	-	-	3.28	-	-	-	3.37	-
doxycycline_MMP1	-	-	-	3.33	-	-	-	-	-	-	-
doxycycline_MMP13	-	-	-	-	-	-	-	2.07	2.8	-	-
doxycycline_MMP3	4.54	-	-	2.5	-	-	-	-	2.69	-	-
doxycycline_TNF	3.98	-	-	-	-	-	-	-	-	-	-
droperidol_DRD2	**-**	**-**	**-**	**-**	**-**	**-**	**-**	**-**	**-**	**3.84**	**-**
drotrecogin alfa_F5	-	-	-	-	-	-	-	-	-	2.82	-
drotrecogin alfa_SERPINE1	-	-	-	5.34	-	2.42	4.6	-	5.03	-	-
epirubicin_TOP2B	-	-	-	-	-	-	-	-	2.19	-	-
erlotinib_EGFR	5.27	-	-	-	-	-	4.93	-	-	-	-
etodolac_PTGS2	8.76	-	-	-	-	-	-	-	-	3.51	-
etoposide_TOP2B	-	-	-	-	-	-	-	-	2.19	-	-
everolimus_MTOR	9.59	-	-	-	-	-	-	-	-	-	4.17
fenoprofen_PTGS2	8.76	-	-	-	-	-	-	-	-	3.51	-
fluphenazine_HTR1B	-	-	1.47	2.01	-	1.92	-	-	-	-	-

pazopanib_KIT	4.42	-	-	-	-	-	-
phentolamine_ADRA2A	-	2.86	-	-	-	-	-
pravastatin_MMP14	3.07	3.41	-	-	-	3.51	2.64
pravastatin_MMP9	4.18	-	-	-	-	-	-
pravastatin_TIMP2	-	2.97	-	-	-	-	-
prochlorperazine_DRD2	2.38	-	-	-	-	-	-
raloxifene_ESR1	4.53	-	-	-	-	4.39	4.37
romidepsin_HDAC1	6.39	2.41	-	-	-	4.90	-
simvastatin_IGF1	2.38	-	-	-	-	-	-
sorafenib_BRAF	-	-	-	-	-	-	3.03
sorafenib_KDR	3.95	-	-	-	-	4.42	-
sorafenib_KIT	4.42	-	-	-	-	-	-
sorafenib_RAF1	5.47	-	-	-	-	-	-
sunitinib_KDR	3.95	-	-	-	-	4.42	-
sunitinib_KIT	4.42	-	-	-	-	-	-
tamoxifen_ESR1	4.53	-	-	-	-	4.39	4.37
teniposide_TOP2A	-	-	-	-	-	4.23	-
thalidomide_NFKB1	5.46	-	-	-	-	-	-
thalidomide_VEGFA	-	-	-	2.49	-	-	-
thioridazine_DRD2	2.38	-	-	-	-	-	-
topotecan_TOP1	-	-	-	-	-	-	-
toremifene_ESR1	4.53	-	-	-	-	4.39	4.37
trazodone_ADRA2A	-	2.86	-	-	-	-	-
valproic acid_HDAC6	7.05	-	-	-	-	3.14	-
valrubicin_TOP2A	-	-	-	-	-	4.23	-
vandetanib_EGFR	-	-	4.84	-	-	-	5.05
vandetanib_KDR	3.95	-	-	-	-	4.42	-
vorinostat_HDAC1	6.39	2.41	-	-	-	4.90	-
vorinostat_HDAC2	-	2.41	-	-	-	-	-
vorinostat_HDAC6	7.05	-	-	-	-	3.14	-
vorinostat_HDAC7	4.03	-	-	-	-	-	-
yohimbine_ADRA2A	-	2.86	-	-	-	-	-

flutamide_PEG10	-	-	1.77	1.75	-	1.78	-	-	-	-	-
fluticasone_PGR	-	-	-	-	-	-	4.63	-	-	-	-
fulvestrant_ESR2	-	-	-	-	-	-	3.93	-	-	-	-
gefitinib_EGFR	5.27	-	-	-	-	-	4.93	-	-	-	-
gefitinib_ERBB2	3.91	-	3.66	-	-	-	-	-	-	4.85	-
guanethidine_ADRA1A	4.45	-	-	-	-	-	-	-	1.65	1.85	-
guanethidine_ADRA1B	3.76	-	-	-	-	-	-	-	-	-	-
guanethidine_ADRA2A	-	-	2.6	-	-	1.96	-	-	-	-	-
guanethidine_ADRA2B	-	-	3.66	-	-	-	1.77	-	-	1.56	-
guanethidine_ADRA2C	-	-	3.37	-	-	-	-	-	-	1.43	-
ibuprofen_PTGS2	8.76	-	-	-	-	-	-	-	-	3.51	-
imatinib_ABL1	**9.16**	-	-	-	-	-	-	-	-	**4.45**	-
imatinib_KIT	-	-	-	-	-	-	4.12	-	-	3.87	-
imatinib_PDGFC	-	-	-	-	-	-	-	1.31	-	-	2.33
imatinib_PDGFRB	-	-	-	3.05	-	-	-	-	-	-	-
ketorolac_PTGS2	8.76	-	-	-	-	-	-	-	-	3.51	-
lapatinib_EGFR	5.27	-	-	-	-	-	4.93	-	-	-	-
lapatinib_ERBB2	3.91	-	3.66	-	-	-	-	-	-	4.85	-
leflunomide_PDGFRB	-	-	-	3.05	-	-	-	-	-	-	-
leflunomide_PTGS2	8.76	-	-	-	-	-	-	-	-	3.51	-
lenalidomide_PTGS2	8.76	-	-	-	-	-	-	-	-	3.51	-
lenalidomide_TNF	3.98	-	-	-	-	-	-	-	-	-	-
lenalidomide_VEGFA	-	-	3.66	2.93	-	-	-	3.35	-	-	-
megestrol_ESR1	6.81	4.87	2.83	-	-	-	-	-	-	-	1.54
meloxicam_PTGS2	8.76	-	-	-	-	-	-	-	-	3.51	-
nabumetone_PTGS2	8.76	-	-	-	-	-	-	-	-	3.51	-
nilotinib_ABL1	9.16	-	-	-	-	-	-	-	-	4.45	-
nilotinib_KIT	-	-	-	-	-	-	4.12	-	-	3.87	-
nilotinib_PDGFRB	-	-	-	3.05	-	-	-	-	-	-	-
octreotide_SSTR2	-	-	-	-	-	-	-	-	-	3.11	-
octreotide_SSTR5	-	-	-	-	-	-	-	2.17	2.01	-	2.56
oxaliplatin_BCL2	-	-	-	-	-	-	-	-	-	4.05	-
oxaliplatin_BCL2L1	**9.59**	-	-	-	-	-	-	-	-	**3.49**	-
panitumumab_EGFR	5.27	-	-	-	-	-	4.93	-	-	-	-
pazopanib_FLT1	-	-	-	3.48	-	-	-	-	-	-	-
pazopanib_KDR	-	-	3.66	4.11	-	-	-	-	-	-	-
pazopanib_KIT	-	-	-	-	-	-	4.12	-	-	3.87	-
pazopanib_PDGFRB	-	-	-	3.05	-	-	-	-	-	-	-
phentolamine_ADRA2A	-	-	2.6	-	-	1.96	-	-	-	-	-
plerixafor_CXCR4	-	-	-	-	-	3.26	3.45	-	-	-	4.89
pravastatin_MMP14	-	3.77	-	-	-	-	3.59	3.67	3.33	-	3.58
pravastatin_TIMP2	-	2.4	-	-	-	-	-	-	-	-	-
prochlorperazine_DRD2	-	-	-	-	-	-	-	-	-	3.84	-
raloxifene_ESR1	6.81	4.87	2.83	-	-	-	-	-	-	-	1.54
raloxifene_ESR2	-	-	-	-	-	-	3.93	-	-	-	-
romidepsin_HDAC1	5.71	-	-	-	-	-	-	-	4.13	3.68	-
simvastatin_RHOA	5.45	2.64	-	3.8	-	-	-	-	3.52	4.08	-
sirolimus_FKBP1A	5.53	-	-	-	-	-	-	-	-	-	-
sirolimus_MTOR	9.59	-	-	-	-	-	-	-	-	-	4.17
sorafenib_FLT1	-	-	-	3.48	-	-	-	-	-	-	-
sorafenib_KDR	-	-	3.66	4.11	-	-	-	-	-	-	-
sorafenib_KIT	-	-	-	-	-	-	4.12	-	-	3.87	-
sorafenib_PDGFRB	-	-	-	3.05	-	-	-	-	-	-	-
sorafenib_RAF1	-	-	-	-	-	-	-	-	**4.26**	-	-

sorafenib_RET	-	-	-	-	1.79	-	3.16	-	-	-	-
sunitinib_CSF1R	-	-	-	-	-	2.8	-	-	3.15	-	-
sunitinib_FLT1	-	-	-	3.48	-	-	-	-	-	-	-
sunitinib_KDR	-	-	3.66	4.11	-	-	-	-	-	-	-
sunitinib_KIT	-	-	-	-	-	-	4.12	-	-	3.87	-
sunitinib_PDGFRB	-	-	-	3.05	-	-	-	-	-	-	-
sunitinib_RET	-	-	-	-	1.79	-	3.16	-	-	-	-
tacrolimus_PPP3CA	-	-	-	-	-	-	-	-	1.35	-	-
tacrolimus_PPP3R1	2.18	-	-	-	-	-	-	-	-	2.51	-
tacrolimus_PPP3R2	2.18	-	-	-	-	-	-	-	-	2.51	-
tamoxifen_ESR1	6.81	4.87	2.83	-	-	-	-	-	-	-	1.54
tamoxifen_ESR2	-	-	-	-	-	-	3.93	-	-	-	-
temsirolimus_MTOR	9.59	-	-	-	-	-	-	-	-	-	4.17
teniposide_TOP2B	-	-	-	-	-	-	-	-	2.19	-	-
thalidomide_FGF2	-	-	-	-	-	-	-	-	-	4.1	-
thalidomide_NFKB1	3.93	-	-	-	-	-	-	-	-	-	-
thalidomide_TNF	3.98	-	-	-	-	-	-	-	-	-	-
thalidomide_VEGFA	-	-	3.66	2.93	-	-	-	3.35	-	-	-
thioridazine_ADRA1A	4.45	-	-	-	-	-	-	-	1.65	1.85	-
thioridazine_DRD2	**-**	**-**	**-**	**-**	**-**	**-**	**-**	**-**	**-**	**3.84**	**-**
tolmetin_PTGS2	8.76	-	-	-	-	-	-	-	-	3.51	-
toremifene_ESR1	6.81	4.87	2.83	-	-	-	-	-	-	-	1.54
trastuzumab_ERBB2	3.91	-	3.66	-	-	-	-	-	-	4.85	-
trazodone_ADRA1A	4.45	-	-	-	-	-	-	-	1.65	1.85	-
trazodone_ADRA2A	-	-	2.6	-	-	1.96	-	-	-	-	-
trazodone_HTR1A	-	-	-	-	-	2.08	1.51	-	-	1.61	-
valproic acid_HDAC6	2.2	-	-	-	-	-	-	-	-	3.71	-
valrubicin_TOP2B	-	-	-	-	-	-	-	-	2.19	-	-
vandetanib_EGFR	5.27	-	-	-	-	-	4.93	-	-	-	-
vandetanib_FLT1	-	-	-	3.48	-	-	-	-	-	-	-
vandetanib_KDR	-	-	3.66	4.11	-	-	-	-	-	-	-
vorinostat_HDAC1	5.71	-	-	-	-	-	-	-	4.13	3.68	-
vorinostat_HDAC2	5.71	-	-	2.05	2.71	2.08	2.11	-	2.55	3.47	-
vorinostat_HDAC3	5.79	-	-	-	-	-	5.24	-	5.84	-	-
vorinostat_HDAC4	2.67	-	-	-	-	-	4.04	-	-	-	-
vorinostat_HDAC6	2.2	-	-	-	-	-	-	-	-	3.71	-
vorinostat_HDAC7	-	-	-	-	-	-	-	3.11	-	-	-
yohimbine_ADRA2A	-	-	2.6	-	-	1.96	-	-	-	-	-
yohimbine_ADRA2B	-	-	3.66	-	-	-	1.77	-	-	1.56	-
yohimbine_ADRA2C	-	-	3.37	-	-	-	-	-	-	1.43	-

D. Parametric Gene Set Enrichment Analysis (PGSEA): Analysis of differences between disease and normal states

Drug	135T	142T	153T	156T	34T	37T	90T
carmustine	-	-	-	2.42	-	-	-
cisplatin	2.80	-	-	-	-	2.89	-
daunorubicin	5.55	-	-	-	-	6.30	-

D'. Parametric Gene Set Enrichment Analysis (PGSEA): Analysis of differences between disease and normal states

Drug	mpnst 02_2	mpnst _94_3	mpnst 95_3b	mpnst _as10	mpnst _as13	mpnst _as15	mpnst _as37	mpnst _as42	mpnst _as45	mpnst 96_2	mpnst 97_6
carmustine	5.06	4.95	2.1	-	-	-	-	-	2.56	5.87	3.25
cisplatin	3.93	3.33	3.07	-	-	-	-	-	-	2.36	2.3
daunorubicin	1.39	2.24	-	-	-	-	-	-	-	-	-
doxorubicin	1.94	1.39	-	1.72	1.52	1.52	1.85	-	2.08	2.89	-
etoposide	1.67	-	1.79	-	-	-	-	-	-	-	-
lomustine	2.18	1.34	2.81	-	-	-	-	-	-	4.1	4.25
paclitaxel	-	-	-	-	-	-	-	-	-	3.75	-
piperazine	1.7	1.4	-	-	-	1.39	-	-	-	-	-

thioguanine	1.53	-	2.9	-	-	-	-	-	1.38	2.14	2.22
thiotepa	1.7	1.4	-	-	-	1.39	-	-	-	-	-
vinblastine	-	-	-	-	-	-	-	-	-	1.71	-

E. Connectivity Map (CMAP): Gene expression changes in response to therapeutics

Drug	135T	142T	153T	156T	34T	37T	90T
biperiden	-	3.14	-	-	3.36	-	-
dapsone	-	-	2.87	-	-	-	-
diphenhydramine	-	2.37	-	-	-	-	-
estradiol	-	-	-	-	-	3.70	-
finasteride	-	-	-	-	2.64		-
fludrocortisone	-	2.72	-	-	2.57		-
gentamicin	-	2.32	-	-	2.52		-
naproxen	-	2.54	-	-	-	-	-

E'. Connectivity Map (CMAP): Gene expression changes in response to therapeutics

Drug	mpnst 02_2	mpnst _94_3	mpnst 95_3b	mpnst _as10	mpnst _as13	mpnst _as15	mpnst _as37	mpnst _as42	mpnst _as45	mpnst 96_2	mpnst 97_6
amitriptyline	-	-	-	1.65	-	-	1.35	-	-	-	-
brinzolamide	-	-	-	-	-	-	1.43	-	-	-	-
bumetanide	-	-	-	-	-	-	2.04	-	-	-	-
clotrimazole	1.35	-	-	-	-	-	-	-	-	-	-
disulfiram	-	-	-	2.1	-	2.13	-	-	1.55	1.95	-
estradiol	-	-	-	-	-	-	2.19	-	-	-	-
etoposide	-	-	-	1.38	2.21	1.78	-	-	1.98	1.49	-
finasteride	-	-	-	-	-	-	2.65	-	-	-	-
fludrocortisone	-	-	-	-	-	-	1.62	-	-	-	-
flutamide	1.52	-	-	-	-	-	-	-	-	-	-
hydrochlorothiazide	-	1.88	-	-	-	-	-	-	-	-	-
iloprost	-	-	-	-	-	-	1.58	-	-	-	-
imatinib	-	-	-	1.44	-	-	-	-	-	-	-
lansoprazole	-	-	-	-	1.43	-	-	-	-	-	-
lomustine	4.4	2.27	-	4.22	2.35	4.7	1.43	-	3.8	1.61	-
naproxen	-	-	-	-	-	-	1.34	-	-	-	-
sirolimus	-	-	4.7	-	-	-	-	-	-	-	-
thioridazine	-	-	2.49	4.7	-	4.7	-	-	-	4.7	-
vinblastine	-	-	-	-	-	-	-	2.9	-	-	-
vorinostat	4.7	-	-	4.7	-	4.7	-	-	4.7	4.7	-

F. Biomarker-based rules - Resistant

Drug	Biomarker	135T	142T	153T	156T	34T	37T	90T
capecitabine	*TYMS*	-	-	-	-	-	-	-
cytarabine	*ADA*	-	-	4.21	-	-	-	-
docetaxel	*ABCB1*	3.48	6.13	-	-	-	-	-
doxorubicin	*ABCC1*	-	12.56	-	-	-	-	-
doxorubicin	*AKR1C3*	-	-	10.00	-	-	-	-
fluorouracil	*TYMS*	-	-	-	-	-	-	-
temozolomide	*MGMT*	-	-	-	4.15	-	-	-
vinorelbine	ABCB1	3.48	6.13	-	-	-	-	-

F'. Biomarker-based rules - Resistant

Drug	Biomarker	mpnst 02_2	mpnst _94_3	mpnst 95_3b	mpnst _as10	mpnst _as13	mpnst _as15	mpnst _as37	mpnst _as42	mpnst _as45	mpnst 96_2	mpnst 97_6
capecitabine	*TYMS*	7.52	3.97	-	16.4	10.6	15.7	-	-	3.33	4.08	-
doxorubicin	*ABCC1*	22.8	40.6	-	-	-	-	-	-	-	-	-
fluorouracil	*TYMS*	7.52	3.97	-	16.4	10.6	15.7	-	-	3.33	4.08	-
temozolomide	*MGMT*	-	2.64	-	-	-	-	-	-	-	-	-

G. Biomarker-based rules - Sensitive

Drug	Biomarker	135T	142T	153T	156T	34T	37T	90T
gemcitabine	*DCK*	-	-	4.39	-	-	-	-
medroxyprogesterone	*PGR*	-	13.86		-	-	-	-

G'. Biomarker-based rules - Sensitive

Drug	Biomarker	mpnst 02_2	mpnst _94_3	mpnst 95_3b	mpnst _as10	mpnst _as13	mpnst _as15	mpnst _as37	mpnst _as42	mpnst _as45	mpnst 96_2	mpnst 97_6
octreotide	*SSTR2*	-	-	-	-	-	-	-	23.7	12.5	-	-
teniposide	*TOP2A*	22.3	4.46	-	15.1	12.3	9.96	4.53	-	20.2	4.47	-
topotecan	*TOP1*	4.3	-	-	-	-	-	-	-	-	2.95	-
