# Supplementary figures and images for: Molecular-guided therapy predictions reveal drug resistance phenotypes and treatment alternatives in malignant peripheral nerve sheath tumors
Source: J Transl Med. 2013 Sep 17;11:213. doi: 10.1186/1479-5876-11-213 (PMC3848568; doi:10.1186/1479-5876-11-213)

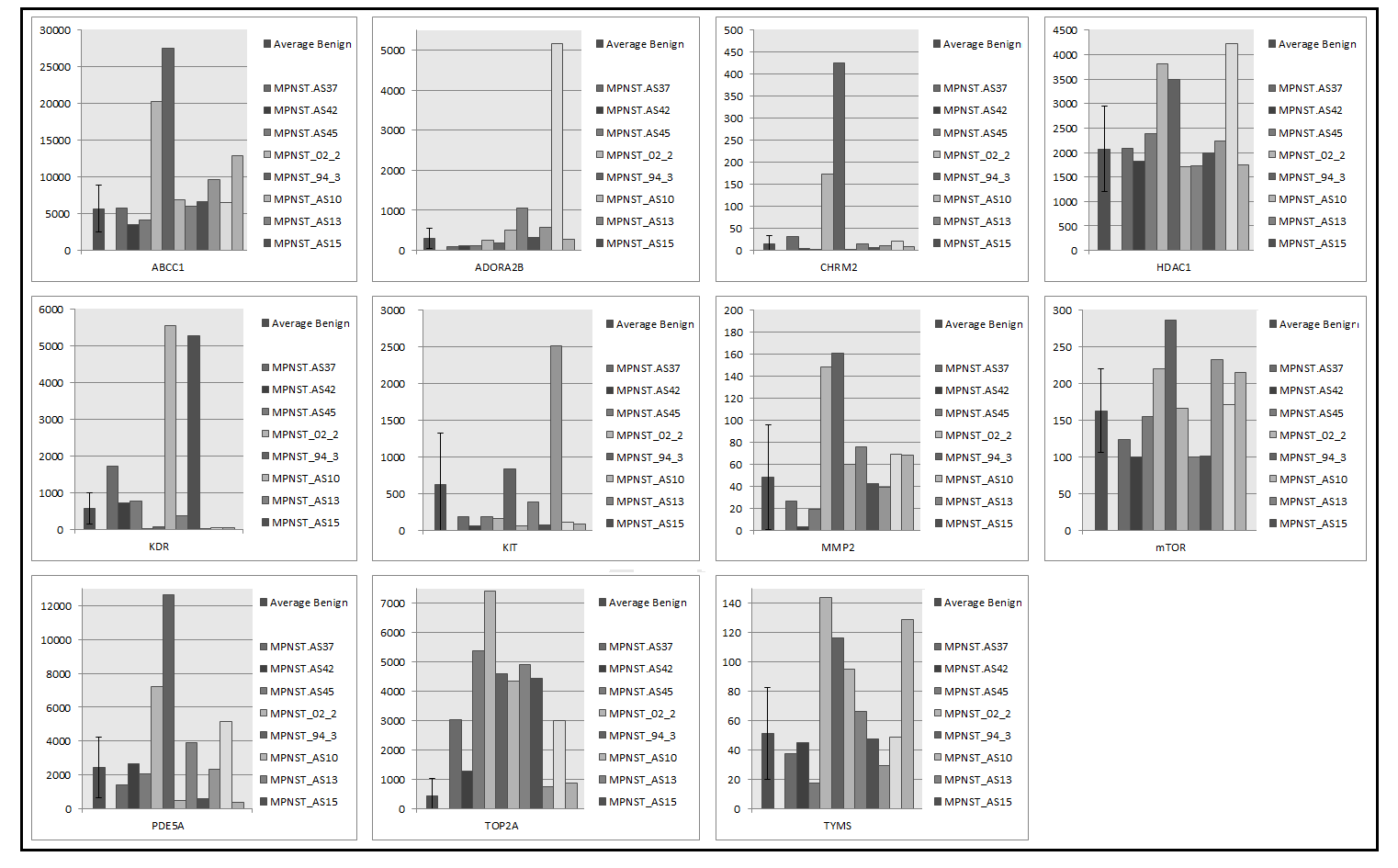

Supplement: Additional file 4 — Relative expression of transcripts related to therapeutic responsiveness scores in the personalized medicine analysis. This figure is an expansion of data presented in Figure 1 for improved clarity. Normalized signal intensity is graphed for ABCC1, ADORA2B, CHRM2, HDAC1, KDR, KIT, MMP2, mTOR, PDE5A, TOP2A, and TYMS with average intensity from benign neurofibromas presented for comparison to the individual MPNST samples as indicated. [file 1479-5876-11-213-S4.png]

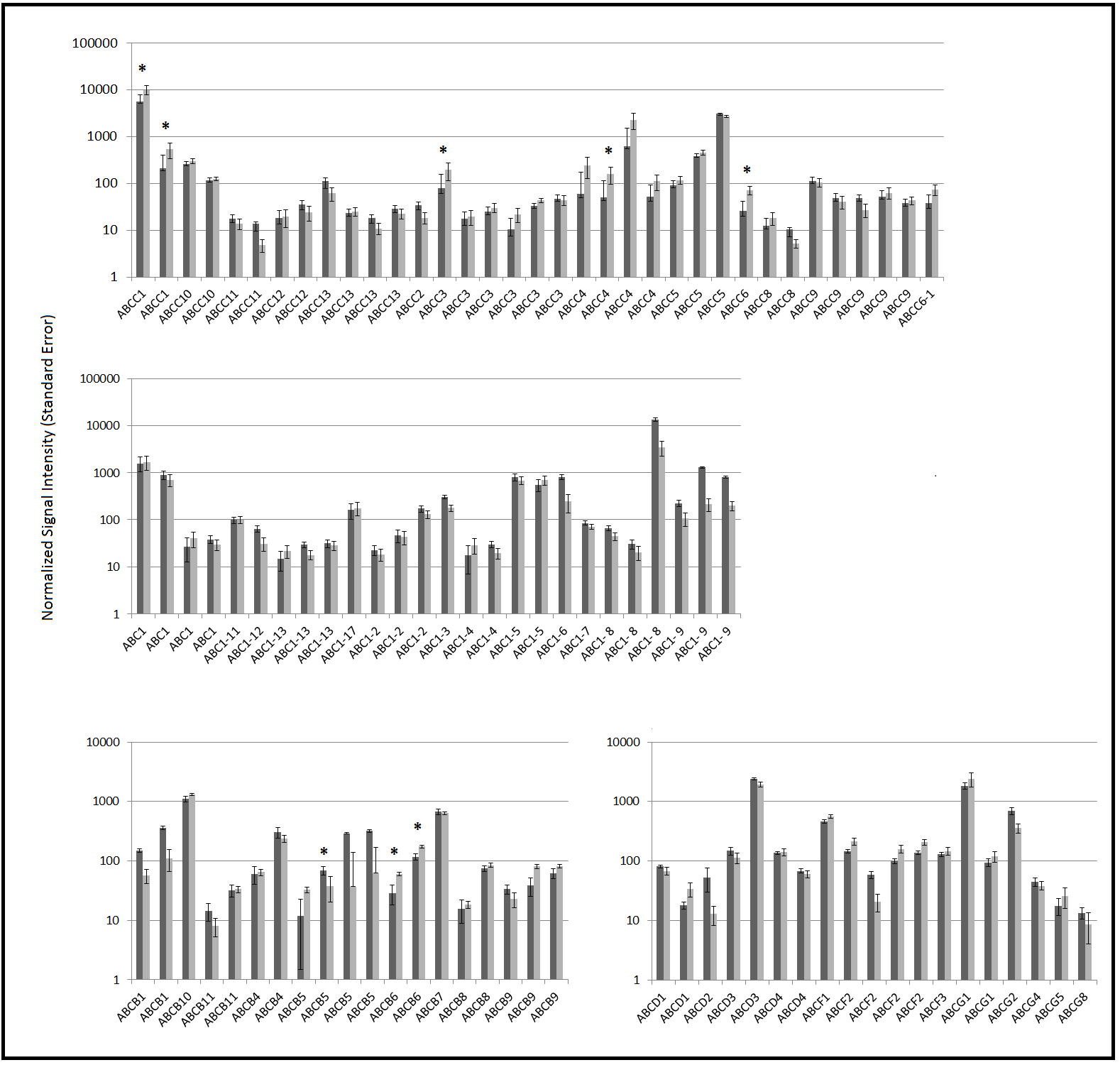

Supplement: Additional file 5 — Relative expression of ABC transporter family genes by microarray. Normalized signal intensity is graphed for all probes for ABCC, ABC1, ABCB, and ABCD-G family transcript expression. Significantly increased transcript levels in MPNSTs compared to plexiform neurofibromas are indicated (*) for p < 0.05. [file 1479-5876-11-213-S5.png]
